# Supplementary material for: Returning to the roots: resolution, reproducibility, and robusticity in the phylogenetic inference of Dissorophidae (Amphibia: Temnospondyli)
Source: PeerJ. 2021 Nov 8;9:e12423. doi: 10.7717/peerj.12423 (PMC8582317; doi:10.7717/peerj.12423)
Supplement: Supplemental Information 1 [file peerj-09-12423-s001.pdf]

Appendices for Gee (2021)

This document contains all text-based appendices associated with the manuscript. Refer to the supplemental material for character matrices (Appendices 4 and 6) and associated tree files (Appendix 7).

Table of contents

Table of contents ... 1
APPENDIX 1. Character list ... 1
Supplemental Table 1. ... 12
Supplemental Table 2. ... 14
APPENDIX 2. Updated scorings to Gee (2020) ... 18
APPENDIX 3. Additional comments on character coding ... 23
APPENDIX 4. Revised character matrix of Gee (2020) ... 25
APPENDIX 5. Updated scorings to Dilkes (2020) ... 26
APPENDIX 6. Revised character matrix of Dilkes (2020) ... 35
APPENDIX 7. Associated .tre files for all analyses ... 35
APPENDIX 8. Summary of previous changes to Schoch's (2012) matrix ... 36
APPENDIX 9. Skull measurement data for olsoniforms ... 39
Supplemental Table 3. ... 39
References ... 42

APPENDIX 1. Character list

This matrix is primarily derived from Gee (2020). As with that matrix, I prefer to organize characters by skeletal region rather than appending new characters to the end of the existing matrix. Therefore, with the addition of several dozen new characters here, characters will not directly align with their numbering system in Gee (2020) after character 6. All new characters, whether they are derived from other matrices or not, are indicated in **blue text**. A guide to correlating characters between differing dissorophid matrices with unique numbering schemes is provided in Supplemental Table 1, and the same for trematopid matrices is given in Supplemental Table 2; these can be found at the end of this appendix. References are given with respect to their character number in the matrices of Polley & Reisz (2011) and Berman *et al.* (2011). These two studies are abbreviated as follows: B = Berman *et al.* (2011); PR = Polley & Reisz (2011). For example, PR-40 refers to character 40 in the matrix of Polley & Reisz (2011): internarial fenestra. All references made with respect to character creation are italicized. Any annotations presented here are related to new or newly reconfigured characters.

Skull roof

1. **Temporal region (lateral expansion)**: lateral margins of skull mostly continuous without prominent inflections (0); temporal region prominently expanded relative to snout region, resulting in a laterally concave inflection along the lateral margin in the suborbital region (1) (*PR-22*).
2. **Ratio of preorbital length to postorbital length**: preorbital length greater than postorbital length by greater than 10% (0); preorbital and postorbital lengths approximately equal (1); postorbital length greater than preorbital length by greater than 10% (2) **(***B-3; PR-51*). [*ordered*]
3. **Suborbital bar height**: greater than 10% of the total midline skull length (0); less than 10% of the total midline skull length (1) **(***PR-52*).
4. **Minimum distance between squamosal embayment and orbital margin**: greater than 25% of the total midline skull length (0); between 10 and 25% of the total midline skull length (1); less than 10% of the total midline skull length (2) **(***PR -53***)**. [*ordered*]
    a. The name is ambiguated from "otic notch" since this term sometimes has specific functional or phylogenetic connotations (e.g., tympanum present).
5. **Prenarial length of skull**: equal to 10% or greater of midline length of skull (0); less than 10% of midline length of skull (1) (*B-27*).
6. **Union between skull table and cheek**: gradual and much less than 90° to produce low, flat skull roof with orbits facing dorsally (0); angle between skull table and cheek sharply defined with cheek oriented nearly vertical to skull table to produce box-like skull roof with orbits facing laterally (1) **(***B-17*).
7. **Posterior skull (lateral profile)**: dorsal margin of the posterior skull roof is confluent with that of the anterior skull, forming a continuous surface (0); dorsal margin is discontinuous, with a distinct inflection point at about the level of the mid-orbit and a steep sloping of the skull from this point anteroventrally (1).
8. **Snout (lateral profile)**: dorsal surface either flat and set at a sloping angle or convexly curved (0), skull roof with prominent inflection anterior to the orbit, producing a horizontal snout (1).
    a. This character is intended to differentiate *Cacops aspidephorus* and *Cacops morrisi* from other dissorophids and from the outgroups. Note that in *Cacops woehri*, state 1 occurs only in small individuals.
9. **Snout (dorsal profile)**: anterior region of skull (around naris) with lateral margin straight in dorsal view (0); laterally convex (1).
    a. This character has been renamed because the lateral expansion does not always occur at the premaxilla-maxilla suture.
10. **Naris (dorsal subdivision):** dorsomedial margin is smoothly curved without partial subdivision by a projection of the nasal (0); nasal projects ventrolaterally to partially subdivide naris (1).
    a. This is a new character that specifically focuses on the condition of some trematopids in which the nasal projects slightly ventrolaterally to partially subdivide the elongated naris from above (e.g., *Acheloma*).
11. **Nasal (lateral margin)**: nasal lateral margin is anteroposteriorly straight such that nasal does not widen anteriorly (0); lateral margin is straight but angled anteroventrally such that nasal widens anteriorly (1); lateral margin is prominently stepped anterior to the prefrontal such that nasal widens anteriorly (2). [*ordered*]

    a. This character is redefined from the one of my previous matrix that I termed 'ventrolateral expansion' (character 9 of Gee, 2020) When the nasal extends laterally beyond the anterior point of the prefrontal, it creates a stepped lateral margin and the impression of a ventrolateral expansion of the element. In trematopids in particular, this can occur either with or without the lateral projection of the nasal into the naris.

12. **Internarial fenestra (fontanelle)**: present (0); absent (1) (*Dilkes, 1990*).
13. **Septomaxilla.** At posterior rim of naris (0); at mid-level of naris, pointing inside (1). (*Schoch, 2012*).
14. **Marginal teeth (caniniform)**: uniform in size (0); caniniform teeth on premaxilla and maxilla (1) (*B-6; PR-41*).
15. **Marginal teeth (bicuspidity)**: the crowns of marginal and palatal teeth are primitively monocuspid (0); the crowns are bicuspid, with the cusps oriented lingually-labially (1) (*Schoch, 2018*).
16. **Marginal teeth (pedicely)**: the crowns of marginal and palatal teeth are conical and continuous with the base (0); the crowns are separated from the base by a poorly or uncalcified zone (pedicely) (1) (*Schoch, 2018*).
17. **Maxillary teeth (count):** greater than 30 positions (0); 30 or fewer (1) (*B-8*).
    a. The numerical ranges are refined to better differentiate most trematopids and some cacopines from all other sampled taxa.
18. **Maxilla (posterior extent)**: maxilla and its dentition extending posterior to the level of the posterior margin of the orbit (0); maxilla extends posterior to the level of the posterior margin of the orbit but dentition terminates at or anterior to the level (1); maxilla terminates at the level of such margin or anterior to it (2) (*PR-26*). [*ordered*]
19. **Maxilla-quadratojugal**: contact present in form of abutting suture, does not divide jugal and maxilla (0); contact present in form of overlapping suture, partially divides jugal and maxilla (1); contact absent (2) (*PR-28*). [*unordered*]
    a. This character has been modified to add an additional state that differentiates a slight abutting contact from a longer overlap by the quadratojugal that partially divides the jugal and the maxilla.
20. **Lacrimal-narial contribution**: lacrimal excluded from naris (0); lacrimal restricted to the posterior border of external naris (1); subnarial process with length less than 50% of the length of the external naris (2); subnarial process with length equal to or greater than 50% of the length of the external naris (3) (*Dilkes, 2020*). [*ordered*]
    a. This character follows Dilkes' modifications to the binary character previously termed "subnarial process of the lacrimal" by Schoch (2012) and derivations thereof, as well as adding a state for a lacrimal-naris separation. The wording of state 1 has been modified slightly.
21. **Lacrimal-orbit**: lacrimal contacts orbital margin (0); lacrimal excluded from orbit (1) (*Anderson et al., 2008*).
22. **Lateral exposure of the palatine (LEP)**: no lateral exposure (0); lateral exposure present (1).
    a. This character replaces (in part) my previous character (character 20 of Gee, 2020) for the circumorbital elements below the orbit. The character was modified because with the synonymy of *Acheloma cumminsi* and *Acheloma dunni*, the

character became parsimony-uninformative, yet the presence of a LEP regardless of configuration is an important apomorphy of xerodromes.

23. **Shape of lateral exposure of the palatine (LEP)**: Elongate exposure along dorsal edge of maxilla (0); enlarged LEP with lateral depression and extends deeply into dorsal border of maxilla (1) (*Dilkes, 2020*).
    a. As a note that may facilitate in the differentiating of character states, state 1 typically produces a stepped dorsal margin in the maxilla such that it is distinctly shorter in height from the anterior orbital margin.
24. **Lateral exposure of the ectopterygoid (LEE)**: absent (0); present (1).
    a. This feature has not been previously included because it is ontogenetically transient in *Cacops morrisi*, disappearing in larger specimens, but it appears that it is a hallmark of maturity in *Acheloma cumminsi* and perhaps *C. aspidephorus* and is of uncertain utility in *Broiliellus reiszi* and *Phonerpeton pricei*. As with the LEP character, this does not differentiate between taxa with differing positions of the LEE; at present, the LEE enters the orbit in all of these taxa except for *A. cumminsi*.
25. **Maxilla-orbit**: maxilla excluded from orbit (0); maxilla enters orbit (1).
    a. This character replaces (in part) my previous character for the circumorbital elements below the orbit (character 20 of Gee, 2020); see rationale for character 23.
26. **Nasals (anterior extent)**: nasals contact premaxillae in a transverse suture at level of anterior margins of external nares (0); nasals extend beyond anterior margins of external nares (1) (*B-5*).
27. **Narial flange**: ventral (inner) side of prefrontal, lacrimal, and nasal smooth (0); inner side of these bones forming complicated bar-like structure (narial flange), permitting contact with antorbital bar (1) (*B-11; PR-13*).
28. **Prefrontal (preorbital region)**: prefrontal extends anteriorly to a level far short of posterior margin of a non-constricted external naris and lateral expansion of the nasal forms almost entire dorsal margin of the naris (0); prefrontal borders posterior third of dorsal margin of posterior subdivision of external naris and posterior half of nasal greatly expanded laterally to contribute about 60 to 70% to dorsal border of external naris (1); prefrontal borders entire or nearly the entire dorsal margin of posterior subdivision of external naris, reducing contribution of nasal to dorsal border of external naris to 40% or less and the lateral expansion of its posterior half to a width equal to or slightly less than anterior half (2) (*B-30*). [*ordered*]
    a. Note that state 0 specifies a non-elongate naris since the prefrontal never contacts the naris unless it is posteriorly elongated (as in trematopids), so any taxon or specimen with a clearly non-elongate naris can be scored, even if the sutures are not preserved in the postnarial region.
29. **Prefrontal (ventral process)**: prefrontal forming simple suture with lacrimal laterally (0); prefrontal underplating lacrimal widely by means of ventral prefrontal process, often abbreviated 'VPP' (1) (*PR-14*).
    a. State 1 is modified to remove mention of 'contacting palatine' because while the underplating of the lacrimal is often reported or figured, whether the process actually contacts the palatine is usually not.
30. **Prefrontal-jugal**: contact present (0); contact absent (1) (*PR-25*).

31. **Prefrontal (ridge)**: prefrontal smooth without prominent expansion along orbital rim (0); prefrontal expanded to form distinct ornamented swelling (1); prefrontal swelling extends anteriorly towards the naris to form a distinct ridge, offsetting the lateral and dorsal surfaces of the snout (2) (*B-2*). [*ordered*]
    a. This character combines the previously used 'prefrontal, postfrontal' character from Berman et al. (2010, 2011), which referred specifically to the presence or absence of ridge-like swellings on these elements, with the 'cranial ridges' character of Schoch (2012), which differentiates between the additional presence or absence of prominent ridges on the snout (found in most dissorophids but not in *Ecolsonia* or *Aspidosaurus*, for example). The character is here restricted to the prefrontal in order to avoid overly complicating the character states. The scorings would not change with the inclusion of the previous specifiers, but some OTUs would have to be left unscored because not all of the specified elements can be assessed.
32. **Prefrontal-postfrontal**: prefrontal and postfrontal firmly sutured, excluding the frontal from orbital margin (0); both elements separated by frontal, at least dorsally (1) (*B-21; PR-21*).
33. **Frontals (anterior extent)**: well beyond level of anterior margins of orbits (0); at approximately level of anterior margins of orbits (1) (*B-4*).
34. **Frontals (midline length)**: exceeded by midline length of parietals (0); exceeds or equals midline length of parietals (*B-28*).
35. **Interorbital width:** Narrow to moderately wide interorbital region in the 0.2–0.24 range (interorbital width/skull length) (0); substantially wider interorbital region (0.27–0.33) (1). (*Schoch, 2018;* originally modified from *Anderson et al., 2008b*).
36. **Postorbital**: narrowing posteriorly, resulting in a more triangular dorsal contour (0); not narrowing, ending bluntly and resulting in a more rectangular dorsal contour (1) (*PR-31*).
    a. Language of the character states has been slightly modified for clarity.
37. **Postorbital-supratemporal:** sutured (0); separated by postfrontal (1). (*Schoch, 2012*).
38. **Parietals (anterior constriction):** parietal tapers gradually with a straight oblique contact with the postfrontal (0); parietal tapers slightly more abruptly with a curved contact with the postfrontal (1); parietal tapers abruptly by a posteromedial expansion of the postfrontal, producing step-like contact (2). [*ordered*]
    a. This character is modified in the same vein as the character regarding the ventrolateral expansion of the nasal and is intended to differentiate the condition seen in some taxa like *Cacops* where the posteromedial portion of the postfrontal forms nearly a right angle with the parietal (state 2) from the condition in most taxa where the tapering is more abrupt and without this prominent step (state 1). Note that this character is frequently polymorphic and varies within a single individual.
39. **Jugal (anterior extent):** irrespective of its lost contact to the lacrimal, the jugal may be either anteriorly long (0); or it may lack the anterior process and end bluntly (1) (*Schoch, 2018*).
40. **Jugal-maxilla**: jugal in abutting contact with maxilla (0); jugal with ventral process that overhangs the maxilla (1).
    a. This feature is found in both *Broiliellus brevis* and *Broiliellus texensis* per Dilkes (2020), and it was also reported in the holotype of *Conjunctio multidens* by

Carroll (1964). Note that this ventral process does not fully divide the maxilla and quadratojugal (character state 18-2 here) but merely obscures the lateral contact between these elements; see Carroll (1964:fig. 10A), for example.

41. **Jugal (eminence)**: jugal ornamented like other roofing elements (0); jugal with developed eminence (1) (*Schoch, 2012*).
42. **Intertemporal**: present (0); absent (1) (*Schoch, 2012*).
43. **Squamosal-supratemporal**: dorsal exposure of suture between squamosal and supratemporal more than half as long as supratemporal itself (0); foreshortened squamoso-supratemporal suture less than half the length of supratemporal (1) (*PR-18*).
44. **Squamosal embayment (exposure)**: embayment well-exposed laterally (0); embayment with reduced lateral exposure of the ventral flange (1) (*PR-43*).
    a. This character is restricted to specifying the autapomorphic condition of *Acheloma* in which the ventral flank of the unornamented flange is mostly obscured in lateral view, and the name is ambiguated from "otic notch" since this term sometimes has specific functional or phylogenetic connotations (e.g., tympanum present).
45. **Squamosal embayment (constriction)**: embayment relatively open, with ventral flank of unornamented flange descending at relatively steep angle (0); embayment dorsoventrally constricted, with ventral flank nearly horizontal (1).
    a. This character is derived in part from my previous character for the exposure of the otic notch (character 37 of Gee, 2020); the other part of the character is retained in character 46 here. This character's state 1 refers to the condition seen in large trematopids (e.g., *Acheloma*, *Ecolsonia*, *Mattauschia*; Milner, 2018:fig. 10) and is essentially equivalent to character 43 of Schoch (2012).
46. **Supratympanic flange**: squamosal continuously ornamented around margin of squamosal embayment (0); squamosal having dorsally exposed and ornamented area (supratympanic flange) stepping abruptly into steeply aligned, poorly ornamented portion (1) (*B-9; PR-19*).
47. **Semilunar flange**: supratemporal without ventral projection into squamosal embayment (0); supratemporal forming marked ventral flange participating in medial bordering of squamosal embayment (1) (*PR-20*).
48. **Semilunar curvature of the squamosal along ventral border of the dorsal unornamented flange of the squamosal embayment**: absent (0); present (1*)* (*PR-49*).
    a. Because the unornamented flange of the squamosal embayment is not always considered a supratympanic flange proper (often restricted to olsoniforms in scorings; e.g., Schoch, 2012), the language of the character name is modified to allow it to be scored for non-olsoniforms.
49. **Supratympanic shelf**: developed ornamentation produces slight overhang (0); flat expansion of roofing elements, with smooth ventral surface, extending laterally over the flange (1) (*Bolt, 1974a*).
    a. Because this feature refers specifically to the supratympanic flange (a feature restricted by some workers to olsoniforms; e.g., Bolt, 1974a; Schoch, 2012), the character states are defined slightly differently from Bolt's three-state character. The derived condition is known from many trematopids (e.g., *Acheloma cumminsi*, *Phonerpeton pricei*, *Tambachia trogallas*); the only taxon in which it is definitively absent is *Ecolsonia cutlerensis*. In *E. cutlerensis* and many

dissorophids, the overhanging by the skull roof is produced by developed ornamentation (irrespective of the type of ornamentation) along the dorsal border, with the ornamentation extending laterally and sometimes ventrally, rather than by a ventrally smooth thin shelf. The former mirrors the condition of most taxa with an squamosal embayment regardless of whether they have a true supratympanic flange. Previous reports of a supratympanic shelf in dissorophids (e.g., Holmes, Berman & Anderson, 2013; Liu, 2018; Dilkes, 2020) are not considered to be homologous with the trematopid condition (following Bolt, 1974a). I note that Daly (1994:8) described both a supratympanic flange and a supratympanic shelf for *Eoscopus lockardi* and compared these features with trematopids, including an express mention of the unornamented ventral surface. A more generalized version of the character referring only to the overhanging of the squamosal embayment could utilize three character states (absent, dissorophid condition, trematopid condition), but here taxa without a supratympanic flange are scored as inapplicable.

50. **Tabular (dorsal exposure)**: tabular narrower than postparietal but reaching almost same surface area as latter (0); tabular distinctly smaller than postparietal, less than 50% of the area of the latter (1) (*B-19; PR-15*).
    a. The name of this character was changed to indicate that the size comparison is only with respect to the surface area exposure on the skull roof, while an admittedly arbitrary relative size threshold was added to the derived state as a crude means of indicating how I scored this character since I did not restrict it to amphibamiforms as in some other studies (e.g., Schoch & Rubidge, 2005).
51. **Tabular (ventral process):** absent (0); short process well-separated from the quadrate (1); well-developed ventral process that closely approaches or that contacts the quadrate (2) (*B-10; PR-45*). [*ordered*]
    a. The character is modified from the previous three-state character of Polley & Reisz (2011), which differentiated the curved tabular horn of *Acheloma* from the more abrupt right angle formed by the horn of dissorophids and *Ecolsonia cutlerensis* but not from this character's state 0 ('short or absent'), and is replaced with the three-state character of Berman et al. (2010, 2011), which does differentiate between 'short' and 'absent' but not between the nature of well-developed tabular horns.
52. **Tabular-squamosal**: elements widely separated by supratemporal (0); squamosal meeting tabular, excluding supratemporal from squamosal embayment (1) (*B-20; PR-16*).
53. **Tabular-exoccipital**: contact absent (0); contact present (1). (*PR-36*).
54. **Postparietal (proportions)**: postparietal of relatively equant proportions, only slightly wider than long (0) or prominently foreshortened with width markedly greater than length (1) (*Schoch, 2012*).
55. **Postparietal-exoccipital**: contact present (0); contact absent (1) (*PR-37*).
56. **Quadrate (dorsal process)**: quadrate having smooth posterodorsal side in plesiomorphic state (0); quadrate with prominent dorsoposterior out-growth: the quadrate process (1) (*PR-2*).
57. **Ornamentation (tubercles)**: raised cranial regions of tubercular ornamentation absent (0); present (1). (*Dilkes, 2020*)

   a. This character was previously the "knobby exostoses" character of Schoch (2012) and derivations thereof and was replaced with the renamed but unaltered character of Dilkes (2020).
58. **Ornamentation (distribution)**: evenly developed throughout (0); varies greatly in intensity (1) (*B-22*).
   a. The original character of Berman et al. (2010, 2011) differentiated regular ornamentation from the pronounced ridges, protuberances, and other topographical features found in many dissorophids. As those features are captured by other characters, this character is based only on the distribution of the primary pattern of ornamentation (subcircular pitting in most taxa). For example, the internarial region of *Cacops morrisi* is distinctly smooth (Gee & Reisz, 2018a), whereas the entire skull of *Acheloma cumminsi* is evenly ornamented (Polley & Reisz, 2011). Note that it does not refer to heterogeneity of ornamentation (e.g., pitting versus grooves), only the spatial distribution of some form of ornamentation.
59. **Ornamentation (dorsal eminences)**: skull table without major raised areas other than ornamenting ridges (0); skull table with elevated eminences on the frontal, parietal, postfrontal, and postparietal (1) (*Schoch, 2012*).

Occiput / Neurocranium

60. **Occiput (dorsal rim):** ornamentation not developed into clear ridge (0); ornament forming marked transverse ridge ('nuchal ridge') (1) (*PR-50*).
   a. The language of the character is modified slightly to indicate that the derived condition is found only in taxa with a clearly offset ridge. Many dissorophines have expanded ornamentation only on the postparietals (and not on the tabulars) that does not form a distinct ridge (although it naturally heightens the offset from the occipital flange), but this is addressed in character 53.
61. **Occiput (posterior margin in dorsal view):** straight or concave (0); biconcave (1) (*B-31*).
62. **Stapes (morphology):** stapes with pronounced dorsodistal curvature (0); stapes without dorsodistal curvature (1) (*PR-24*).
63. **Stapes (stapedial foramen)**: absent (0); present (1) (*PR-46*).
64. **Opisthotic-prootic**: co-ossified (0); distinct and not co-ossified (1).
65. **Sphenethmoid:** ossified (0); unossified (1) (*Anderson et al., 2008a*).

Palate

66. **Parasphenoid (dentition):** basal plate of parasphenoid bearing shagreen of small teeth (denticles) anteromedially (0); plate entirely smooth (1) (*B-33 [in part]; PR-4*).
67. **Parasphenoid (denticle field)**: parasphenoid denticle field well established, with triangular outline and with apex reaching onto base of cultriform process (0); denticle field greatly expanded anteriorly to cover most of the cultriform process (1) (*B-33 [in part]; PR-5*).
68. **Parasphenoid (basal plate)**: basal plate approximately square, as long as wide (0); basal plate wider than long, but greatest width less than twice the length (1); basal plate at least twice as wide as long (2) (*PR-6*). [*ordered*]

    a. This character is modified to add an intermediate state because many dissorophids have a basal plate that is distinctly longer than it is wide, but that is not twice as wide as it is long. In this analysis, state 2 is found only in *Doleserpeton annectens* and *Broiliellus reiszi*, but it is found more widely among amphibamiforms.

69. **Parasphenoid (muscle scars)**: the posterior and posterolateral parts of the parasphenoid plate bear gentle depressions or other types of muscle attachments (0); or they house deep pockets for such attachments (1) (*Schoch, 2012*).
70. **Parasphenoid (foramina for the carotid arteries):** widely separated, on basal plate of parasphenoid (0); closely spaced, on basal plate near base of cultriform process (1); closely spaced, on process (2) (*Schoch, 2012*). [*ordered*]
    a. This character is modified to add an additional state that differentiates closely spaced foramina on the basal plate near the base of the process (e.g., *Aspidosaurus binasser*; *Pasawioops mayi*) from those that are actually on the process (e.g., *Cacops morrisi*, *Cacops woehri*).
71. **Parasphenoid (cultriform process):** moderately wide and flat on ventral side (0); throughout thin and round in cross-section (1) (*Schoch, 2012*).
72. **Basicranial articulation:** open (0); sutured or fused (1) (*B-32*).
73. **Choana**: narrow, forming elongated oval with parallel lateral and medial margins (0); expanded anteromedially (1) (*B-18*).
74. **Vomerine depression**: ventral surface of vomers flat (0); single unpaired depression (intervomerine fossa) in anterior portion of vomers that may or may not house an opening (1) (*PR-3*).
75. **Vomer (posterolateral ramus)**: vomer posterolaterally truncated without process extending along medial margin of the palatine (0); vomer extending posteriorly along medial margin of the palatine (1) (*PR-32*).
76. **Vomer (dentulous ridge)**: with (0) or without (1) a toothed, raised crest running anteroposteriorly and lying mesial to the choana (*PR-33*).
77. **Vomer (median septum)**: absent (0); present (1) (*PR-44*).
78. **Vomer (denticle field)**: vomer covered with more or less dense shagreen of teeth in addition to fang pair (0); shagreen confined to juvenile stages and /or absent throughout ontogeny (1) (*PR-7*).
79. **Vomer (fangs)**: vomer lacking fangs in its medial portion, outside lateral tooth arcade, but having smaller accessory teeth in that region (0); vomer with additional fang pairs posterior to mid-vomerine depression (1) (*PR-8*).
80. **Palatine, ectopterygoid**: palatine and ectopterygoid much wider than maxilla (0); palatine and ectopterygoid reduced to narrow struts not wider than adjoining maxilla (1) (*B-15; PR-11*).
81. **Interpterygoid vacuity**: interpterygoid vacuity roundish or oval in outline (0); interpterygoid vacuity greatly expanded laterally at mid-level (1) (*B-14; PR-12*).
82. **Pterygoid-vomer**: suture between pterygoid (palatine ramus) and vomer present (0); pterygoid contacting only posterior-most portion of palatine and lacking suture with vomer (1) (*B-13; PR-9*).
83. **Pterygoid (transverse flange)**: palatine ramus of pterygoid merging continuously into basipterygoid ramus (0); palatine ramus broadening abruptly to form well-developed transverse flange (1) (*PR-10*).

Mandible

84. **Jaw articulation:** posteriormost margin of quadrate at level posterior to the occipital margin of the skull at the midline; (0); in line with the occipital margin (1); anterior to the occipital margin (2) (*PR-38*). [*ordered*]
    a. This character is redefined with respect to the landmarks. Several taxa have been scored for this character despite the exoccipitals being unknown (e.g., *Cacops woehri* in which both the exoccipital and the quadrate are unknown), so the revised character states reflect the historic practice of using the occipital margin as a proxy for the occiput (since most temnospondyls have vertical occiputs). This also disambiguates the position of the jaw articulation, which is a region rather than a distinct position.
85. **Prearticular (medial inflection):** absence of a medial inflection of the prearticular along the medial rim of the adductor fossa (0); inflection present (1) (*PR-42*).
86. **Inframeckelian fossa**: Bordered only by prearticular and angular (0); bordered by prearticular, angular, and postsplenial (1) (*Dilkes, 2020*).
87. **Angular (ventral edge in lateral view)**: dermal sculpturing similar to remainder of angular (0); dermal sculpturing enlarged to form a keel-like projection at the posterior corner (1) (*Dilkes, 2020*).

Postcrania

88. **Osteoderms (distribution):** absent (0); present in single row along vertebral column (1); present, forming dorsal covering over trunk (2). [*unordered*]
    a. This character is substituted for the previous binary "dorsal ossifications" character of Gee (2020), which was derived from character 25 (carapace) of Berman et al. (2010); the new character follows that used in more general temnospondyl analyses (*Schoch, 2013*). State 2 is found in *Anconastes vesperus* and *Ecolsonia cutlerensis* (Berman, Reisz & Eberth, 1985, 1987).
89. **Osteoderm width**: less than twice as wide as it is long (0); at least twice as wide as it is long (1) (*Dilkes, 2020*).
    a. This character follows Dilkes (2020) in restricting it to two states without a specified point of comparison (e.g., associated vertebra) because one is not always available for many specimens. The 'twice as long' threshold is undoubtedly arbitrary but does capture the differentiation utilized in previous studies (e.g., *Broiliellus arroyoensis*, *Broiliellus texensis*, and *Dissorophus multicinctus* have wide osteoderms; all other dissorophids have narrow osteoderms).
90. **Osteoderm series**: Single series throughout (0); double series associated with at least anterior vertebrae (1). (*Dilkes, 2020*).
91. **Internal osteoderms**: Sutured or fused to vertebrae (0); unfused with ventral flange (1). (*Dilkes, 2020*).
    a. Note that this character is also scored for taxa with only a single series.
    b. State 0 is adjusted to "sutured or fused" to reflect that several taxa are known from material in which fusion is apparent anteriorly but not posteriorly, likely reflecting ontogenetic immaturity in the axial development.
92. **External osteoderms**: Absence of ventral flange (0); presence of ventral flange (1). (*Dilkes, 2020*).

    a. Note that this character is considered inapplicable for taxa with only a single series.

93. **Ventral flanges:** All flanges between neural spines (0); flanges of anterior internal osteoderms extend along anterior and posterior sides of neural spine (1). (*Dilkes, 2020*).
94. **Cleithrum (head)**: head of cleithrum aligned along anterior rim of scapula (0); cleithrum with posterodorsally enlarged head, wrapping around scapula dorsally (1) (*Schoch, 2018*).
95. **Scapula:** scapula forming low or moderately high element, depending on degree of ossification, about two times longer than wide (0); scapula dorsally much extended, being three to four times longer than wide (1) (*Schoch, 2018*).
96. **Humerus (supinator process):** present in adults (0); absent throughout ontogeny (1) (*B-26)*.
97. **Humerus (deltopectoral depression)**: absent (0); present (1).
    a. This circular socket-like feature is found in *Diploseira angusta* and *Dissorophus multicinctus* (Dilkes, 2020).
98. **Femur (adductor crest)**: crest deflected at the proximal termination (0); crest transversely expanded at proximal termination with bifurcation or depression (1); crest terminates in distinct, ventrally convex protuberance (2). [*unordered*]
    a. Although some workers have referred to the entire expanded proximal region as the trochanter (e.g., Sullivan, Reisz & May, 2000), others restrict the trochanter to the anterior edge of this region (e.g., Pawley & Warren, 2006); therefore, this feature is ambiguated to refer to the proximal terminus of the adductor crest. State 1 is like that found in *Acheloma* (e.g., Williston, 1909; Olson, 1941) or *Parioxys bolli* (Carroll, 1964b), and state 2 is like that found in terrestrially capable amphibamiforms (most of the historical 'amphibamids'; e.g., Daly, 1994; Sigurdsen & Bolt, 2010).
    b. It should also be noted that there often appears to be a difference in the deflection of the terminus from the rest of the crest (e.g., compare cf. *Acheloma* and cf. *Cacops* of Sullivan, Reisz & May, 2000), but this is directly related to whether or not the region is expanded (deflected in the absence of expansion versus straight in the presence of expansion), which is why this is not a separate character.
99. **Interclavicle**: quadrangular to pentagonal, subequal in width and length (0); rhomboidal, longer than it is wide (1); longer than it is wide and with a prominently offset posterior stylus (2) (*Schoch, 2012*, clarified by *Liu, 2018*). [*ordered*]
    a. States 0 and 1 were reversed from the original character. Some edits to terminology of character states have also been made.
100. **Clavicle ornamentation**: ornamented with pitting or grooves similar to the skull (0); clavicle smooth (1).
101. **Ilium (dorsal process):** process high and slender, distally not wider than shaft (0); or short and stout, not higher than base is wide, with broadened dorsal end (1) (*Schoch, 2018*).
102. **Atlas (neural arch)**: notochordal canal dorsally open, with two separated prongs (0); notochordal canal dorsally closed with prongs sutured or fused (1) (*Anderson et al., 2008a*).
103. **Presacral intercentra:** wedge-shaped (0); dorsally enlarged, forming a nearly to fully enclosed ring (1) (*Schoch, 2018*).

a. Character renamed to indicate that this is specifically for presacral trunk vertebrae.

104. **Presacral pleurocentra:** confined to the dorsal portion of the vertebral centrum (0); reaching ventrally down the flanks (1); forming short closed rings (2); forming elongate cylinders (3) (*Schoch, 2018*). [*ordered*]

a. Character renamed to indicate that this is specifically for presacral vertebrae.

105. **Trunk ribs:** moderately long (> length of three vertebrae) (0); short (< length of three vertebrae) (1) (*Schoch, 2018*).

106. **Anterior trunk ribs:** anterior trunk ribs with widened ends (0); ribs are simple rods without distal expansion (1). (*Schoch, 2018*).

107. **Presacral count:** 26 or more presacral vertebrae (0); 23-25 (1); 22 or fewer (2) (*Schoch, 2018*). [*ordered*]

a. This character is modified from the previous version (a binary character with states of '24' or 'less than 24') and is more along the lines of the version used in broader temnospondyl analyses (e.g., Schoch, 2013), though with the three states slightly redefined to capture all possible presacral counts (previously, any taxon with 21, 22, 27, or 28 presacral positions was not scorable).

108. **Ribs (uncinate processes)**: absent on trunk ribs (0); straight, spike-like processes present (1).

109. **Sacral rib (count)**: 1 (0); 2 (1).

a. At present, state 1 is restricted to *Parioxys bolli* among dissorophids (Carroll, 1964b, assuming this taxon is indeed a dissorophid), although it may also occur in the unscored *Parioxys ferricolus* (Moustafa, 1955). While *Cacops aspidephorus* has long been characterized as also having two sacral ribs (Williston, 1910), Dilkes (2009) argues that it is not possible to assess the number of these ribs given the heavy reconstruction of the sacral region in the holotype.

Supplemental Table 1.

Character correlation key showing the equivalent characters between different dissorophid-focused studies, benchmarked to Dilkes (2020). Abbreviations: D20, Dilkes (2020); F12, Fröbisch & Reisz (2012); G21, Gee et al. (2021); H13, Holmes, Berman & Anderson (2013); L18, Liu (2018); M13, Maddin et al. (2013). Note that Fröbisch & Reisz (2012) is not included here, but all 53 characters are equivalent to the first 53 characters of Schoch (2012). Because this table is benchmarked against Dilkes (2020) as the most recent version of the widely propagated Schoch (2012) matrix, not all characters of Gee et al. (2021) or this study are listed; these have no equivalents in other studies. Black boxes indicate no equivalent for the characters that are listed. Characters are colored from red (low character number) to green (high character number).

Character	F12	S12	S13	H13	M13	L18	D20	G21	This study
Lateral exposure of the palatine	1	1	1	1	1	1	1	1	22
Shape of LEP							2		23
Maxilla-orbit				2		2	3		25
Dorsal quadrate process	2	2	2	3	2	3	4	2	56
Vomerine depression	3	3	3	4	3	4	5	3	74
Parasphenoid dentition	4	4	4	5	4	5	6	4	66
Parasphenoid denticle field	5	5	5	6	5	6	7	5	67

Parasphenoid basal plate	6	6	6	7	6	7	8	6	68
Vomerine denticle field	7	7	7	8	7	8	9	7	78
Vomerine fangs	8	8	8	9	8	9	10	8	79
Pterygoid-vomer	9	9	9	10	9	10	11	11	82
Pterygoid flange	10	10	10	11	10	11	12	12	83
Palatine, ectopterygoid	11	11	11	12	11	12	13	68	80
Interpterygoid vacuity	12	12	12	13	12	13	14	15	81
Narial flange	13	13	13	14	13	14	15	20	27
Prefrontal process	14	14	14	15	14	15	16	21	29
Tabular size	15	15	15	16	15	16	17	22	50
Tabular-squamosal	16	16	16	17	16	17	18	23	52
Squamosal-supratemporal suture	17	17	17	18	17	18	19	25	43
Supratympanic flange	18	18	18	19	18	19	20	26	46
Semilunar flange	19	19	19	20	19	20	21	27	47
Prefrontal-postfrontal	20	20	20	21	20	21	22	29	32
Interorbital width	21	21	21	22	21	22	23	58	35
Palpebral ossifications		22	22	23	22	23	24	69	
Stapes	23	23	23	24	23	24	25	31	62
Prefrontal-jugal	24	24	24	25	24	25	26		30
Maxilla dentition	25	25	25	26	25	26	27	70	9
Skull outline	54	26	26	27	26	27	28	71	9
Septomaxilla	27	27	27	28	27	28	29	72	12
Parietal width	28	28	28	29	28	29	30	73	
Postparietal length	29	29	29	30	29	30	31	24	54
Postorbital	30	30	30	31	30	31	32	74	36
Vomer (posterior projection)	31	31	31	32	31	32	33	75	75
Vomer (tooth row)	32	32	32	33	32	33	34	76	76
Palatine-interpterygoid vacuity	33	33	33	34	33	34	35		
Cultriform process	34	34	34	35	34	35	36	77	71
Exoccipital-tabular	35	35	35	36	35	36	37		53
Exoccipital-postparietal	36	36	36	37	36	37	38	78	55
Position of jaw articulation	37	37	37	38	37	38	39	65	84
External narial opening	38	38	38	39	38	39	40	79	
Internarial fenestra	39	39	39	40	39	40	41	80	11
Marginal teeth of upper jaw	40	40	40	41	40	41	42	81	13
Prearticular	41	41	41	42	41	42	43	82	85
Ventral border of otic notch	42	42	42	43	42	43	44	56	45
Median vomerine septum	43	43	43	44	43	44	45	83	77
Tabular process	44	44	44	45	44	45	46	84	51
Stapedial foramen	45	45	45	46	45	46	47	85	63
Tubercular ornamentation	46	46	46	47	46	47	48	59	57
Lacrimal-naris	47	47	47	48	47	48	49	86	20
Squamosal	48	48	48	49	48	**49**	50	87	48
Dorsal rim of occiput	49	49	49	50	49	50	51	88	60
Preorbital-postorbital length ratio	50	50	50	51	50	51	52	89	2

Suborbital bar height	51	51	51	52	51	52	53	90	3
Minimum distance of otic notch-orbit	52	52	52	53	52	**53**	54	91	4
Basipterygoid articulation		53	53	54	53	54	55	92	72
Postorbital-supratemporal		54	54	55	54	55	56	64	37
Intertemporal		55	55	56	55	56	57	61	42
Osteoderms		56	56	57	56	57	58	93	88
Osteoderm width		57	57	58	57	58	59	94	89
Cranial ridges		58	58	59	58	59	60	95	31
Ilium (dorsal process)	53	59	59	60	59	60	61	39	100
Parasphenoid plate		60	60	61	60	61	62	96	68
Carotid artery		61	61	62	61	62	63	49	70
Tabular horn		62	62	63	62	63	64	46	
Jugal		63	63	64	63	64	65	97	41
Parasphenoid: muscle scars		64	64	65	64	65	66	98	69
Pointed snout		65	65	66	65	66	67	99	
Interclavicle		66	66	67	66	67	68	38	98
Supinator process		67	67	68	67	68	69	36	95
Entepicondylar foramen		68	68	69	68	69	70	67	
Dorsal eminences		69	69	70	69	70	71	100	59
Ventral edge of angular		70	70	71	70	71	72		87
Osteoderm series							73		90
Internal osteoderms							74		91
External osteoderms							75		92
Association of flange with neural spines							76		93
Inframeckelian fossa							77	51	86

Supplemental Table 2.

Character correlation key showing the equivalent characters between different trematopid-focused studies, benchmarked to this study. Abbreviations: B10, Berman et al. (2010); B11, Berman et al. (2011); G2, Gee (2020); P11, Polley & Reisz (2011). Benchmarking is to this study since no one matrix has been widely propagated like in the study of dissorophids. Black boxes indicate no equivalent for the characters that are listed. Characters are colored from red (low character number) to green (high character number).

Character	B10	B11	P11	G20	This study
Temporal region (lateral expansion)	23	23	22	1	1
Preorbital-postorbital length ratio	3	3	51	2	2
Suborbital bar height			52	3	3
Minimum distance of otic notch-orbit			53	4	4
Prenarial length		27		6	5
Skull table-cheek	17	17		7	6
Posterior skull (lateral profile)					7
Snout (lateral profile)					8
Snout (dorsal profile)			27	17	9

Naris (elongation)	1	1	39	8	
Naris (dorsal subdivision)				9	10
Nasal (lateral margin)				9	11
Internarial fenestra	7	7	40	10	12
Septomaxilla				11	13
Marginal teeth (caniniform)	6	6	41	12	14
Marginal teeth (bicuspidity)				13	15
Marginal teeth (pedicely)				14	16
Maxillary teeth (count)	8	8		15	17
Maxilla (posterior extent)			26	16	18
Maxilla-quadratojugal			28	18	19
Lacrimal-naris				19	
Lacrimal (narial contribution)			48	19	20
Lacrimal-orbit				19	21
LEP (presence/absence)	12	12	1	20	22
LEP (shape)					23
LEE					24
Maxilla-orbit				20	25
Nasals (anterior extent)	5	5		21	26
Narial flange	11	11	13	22	27
Prefrontal (preorbital region)		30		23	28
Prefrontal (ventral process)			14	24	29
Prefrontal-jugal			25	25	30
Prefrontal (ridge)	2	2		26	31
Prefrontal-postfrontal	21	21	21	27	32
Frontals (anterior extent)	4	4		28	33
Frontals (midline length)		28		29	34
Interorbital width				30	35
Postorbital			31	32	36
Postorbital-supratemporal				32	37
Parietal width		29	29	34	38
Jugal (anterior process)				35	39
Jugal-maxilla					40
Jugal (eminence)					41
Intertemporal					42
Squamosal-supratemporal			18	36	43
Squamosal embayment (exposure)				36	44
Squamosal embayment (constriction)			43	37	45
Supratympanic flange	9	9	19	38	46
Semilunar flange			20	39	47
Squamosal			49	40	48
Supratympanic shelf					49
Tabular size	19	19	15	41	50
Tabular process	10	10	45	42	51

Tabular-squamosal	20	20	16	43	52
Exoccipital-tabular			36	44	53
Postparietal length	16	16	17	45	54
Postparietal proportions			30	45	54
Exoccipital-postparietal			37	46	55
Dorsal quadrate process			2	47	56
Tubercular ornamentation			47	48	57
Ornamentation (distribution)	22	22		49	58
Dorsal eminences				50	59
Dorsal rim of occiput			50	51	60
Occiput contour		31		52	61
Stapes			24	53	62
Stapedial foramen			46	54	63
Opisthotic-prootic					64
Sphenethmoid				55	65
Parasphenoid dentition		33	4	56	66
Parasphenoid denticle field		33	5	57	67
Parasphenoid basal plate			6	58	68
Parasphenoid (muscle scars)					69
Carotid arteries				59	70
Cultriform process			35	60	71
Basicranial articulation		32		61	72
Choana	18	18		62	73
Vomerine depression			3	63	74
Vomer (posterior projection)			32	64	75
Vomer (tooth row)			33	65	76
Median vomerine septum			44	66	77
Vomerine denticle field			7	67	78
Vomerine fangs			8	68	79
Palatine, ectopterygoid	15	15	11	69	80
Interpterygoid vacuity	14	14	12	70	81
Ectopterygoid				71	
Pterygoid-vomer	13	13	9	72	82
Palatine-interpterygoid vacuity			34	72	82
Pterygoid flange			10	73	83
Position of jaw articulation			38	74	84
Prearticular	24	24	42	75	85
Inframeckelian foramen					86
Angular (ventral edge in lateral view)					87
Dorsal ossifications	25	25		76	88
Osteoderm width					89
Osteoderm series					90
Internal osteoderms					91
External osteoderms					92
Ventral flanges					93

Cleithrum (head)				77	94
Scapula				78	95
Humerus (supinator process)	26	26		79	96
Humerus (deltopectoral depression)					97
Femur (adductor crest)					98
Interclavicle					99
Clavicle ornamentation					100
Ilium (dorsal process)				80	101
Atlas (neural arch)					102
Presacral intercentra				81	103
Presacral pleurocentra				82	104
Trunk ribs				83	105
Anterior trunk ribs				84	106
Presacral count (reduction)				85	107
Ribs (uncinate processes)					108
Sacral rib (count)					109
Snout shape				5	
Palpebral ossifications			23	31	

APPENDIX 2. Updated scorings to Gee (2020)

This appendix summarizes scoring changes (updates or corrections) made from my previous matrix (Gee, 2020) with justifications wherever appropriate. Note that this does not include scores that were revised as part of a revision of a character or one of its states. Updated scores refer to previous unscored cells that are newly scored based either on new data or upon re-examination of scoring for the entire character or previously scored cells that are now scored as polymorphic (inclusive of the original scored state). Revised scores are based on a reconsideration of scoring approach or the specific condition. Corrected scores are assumed to have been typographic unless otherwise stated. Character numbering is of the revised matrix.

Acheloma cumminsi

- Character 38 (parietals [anterior constriction]): 1 → 1&2: this is an updated score based on the polymorphism observed in the holotype of "*Acheloma dunni*" by Polley & Reisz (2011).

Phonerpeton pricei

- Character 62 (stapes [morphology]): 1 → ?: this is a correction; the stapes is not sufficiently exposed or preserved to be characterized.
- Character 66 (parasphenoid [dentition]): 0 → 1: this is a correction based on Dilkes' (1990) comment that there is no apparent dentition on the basal plate, whereas denticles are preserved on other regions of the palate.
- Character 109 (anterior trunk ribs): 0 → 1: this is a correction.

Rotaryus gothae

- Character 9 (snout [dorsal profile]): 1 → 0: this is a correction; the very slight flexure is considered too minor be scored for the derived state compared to other taxa.
- Character 50 (tabular [dorsal exposure]): 0 → ?: this is an update based on the specification of "size" as surface area; distortion of the holotype does not permit confident comparison of the tabular and the postparietal, as evidenced by the marked asymmetry of the postparietals.

Fedexia striegeli

- Character 36 (postorbital): 0&1 → 0: this is a revision informed by the close examination of scores for dissorophids; the left postorbital does not end in a sharp point, but it does taper to a rounded terminus rather than being squared off like in some dissorophids.

Actiobates peabodyi

- Character 34 (frontals [midline length]): 0 → 1: this is a correction.

Anconastes vesperus

- Character 34 (frontals [midline length]): ? → 0: this is an updated score.
- Character 38 (parietals [anterior constriction)]: ? → 1: this is an updated score.
- Character 84 (jaw articulation): 2 → 0: this is a correction.

Tambachia trogallas

- Character 6 (skull table-cheek): 1 → ?: this is a correction; the skull is too flattened to be reliably scored.
- Character 18 (maxilla [posterior extent]): ? → 0/1: this is an updated scoring for partial uncertainty. While the posterior extent of the dentition is unclear, the maxilla itself

definitively extends past the posterior margin of the orbit, thereby excluding state 2 from consideration.

- Character 29 (prefrontal [ventral process]): 1 → ?: this is a correction based on Sumida et al.'s (1998) comment that no ventral process is evident.
- Character 36 (postorbital): 0 → 1: this is a revision informed by the close examination of scores for dissorophids; the left postorbital does have a small projecting angle along the posterior margin, but it does not taper to a sharp terminus like in other taxa.
- Character 48 (tabular [dorsal exposure]): 0 → ?: this is an updated score, informed by the specification of "size" as surface area and by the uncertain anteromedial margin of the postparietals, which hypothetically could have extended farther forward than the preserved anterolateral margins.

Ecolsonia cutlerensis

- Character 36 (postorbital): 0&1 → 1: this is a revised score based on the close examination of scores for dissorophids; the postorbital in CM 41703 does taper posteriorly, but it does not form a narrow projection.
- Character 43 (supratemporal-squamosal): 0 → 0&1: this is an updated score based on the polymorphism observed by Berman, Reisz & Eberth (1985:fig 5A).
- Character 105 (trunk ribs): 1 → 0: this is a correction.

Mordex calliprepes

- Character 18 (maxilla [posterior extent]): 0 → 0/1: this is a revised scoring on the basis that while the maxilla terminates posterior to the orbit, the termination of the tooth row cannot be confidently determined (partial uncertainty between states 0 and 1).
- Character 62 (stapes [morphology]): 1 → ?: this is a correction.

Mattauschia laticeps

- Character 106 (anterior trunk ribs): 0 → ?: this is a revised score on the basis that proportion-based postcranial characters should not be scored from the small holotype.

Broiliellus brevis

- Character 19 (maxilla-quadratojugal): ? → 0/1: this is an updated score that accounts for partial uncertainty; these elements contact as seen in ventral view, but their suture in lateral view is obscured by the overhanging jugal.
- Character 26 (nasals [anterior extent]: 0 → ?: this character was scored in error based on Carroll's (1964) inferred position of the premaxilla-nasal suture.
- Character 35 (interorbital width): 0 → 1: this is a correction.
- Character 36 (postorbital): 0&1 → 1: this is an updated score based on a comparison with other dissorophids; while the posterior margin has a slight angle, it does not taper sharply or form a prominent posterior process.
- Character 61 (occiput [posterior margin]): 1 → ?: this character was changed to being unscored here because it is not possible to determine the contour of the dorsal margin of the occiput based on Carroll's (1964a) illustrations alone (fig. 9A therein).
- Character 63 (stapedial foramen): 0 → ?: this character was changed to being unscored here because it is not possible to determine the presence or absence of a foramen based on Carroll's (1964a) illustrations and single sentence description alone (p. 197, fig. 10A therein).
- Character 105 (trunk ribs): 1 → 0: this is a correction.

Dissorophus multicinctus

- Character 35 (interorbital width): 0 → 1: this is a correction.
- Character 36 (postorbital): 0 → 0&1: this is an updated score based on Dilkes (2020).
- Character 60 (occiput [dorsal rim]): 1 → 0: this is a correction based on the distinction between dorsal protuberances and the discrete nuchal ridge seen in cacopines.
- Character 81 (interpterygoid vacuity): 0 → 1: this is a correction.

Cacops woehri

- Character 18 (maxilla [posterior extent]): 1 → 0: this is a correction. The derived condition occurs in the immature holotype but not in the larger referred specimen (OMNH 79338) described by Gee, Bevitt & Reisz (2019).
- Character 36 (postorbital): 0&1 → 1: this is an updated score based on a comparison with other dissorophids; while the posterior margin has a slight angle, it does not taper sharply or form a prominent posterior process.
- Character 43 (squamosal-supratemporal): 1 → 0: this is a correction.
- Character 62 (stapes [morphology]): 1 → ?: this is a correction.
- Character 63 (stapedial foramen): 1 → ?: this is a correction.

Pasawioops mayi

- Character 61 (occiput [posterior margin]): ? → 1: this is an updated score.

Eoscopus lockardi

- Character 2 (preorbital-postorbital length): 2 → 1: this is a correction.
- Character 46 (supratympanic flange): ? → 1: this is an updated score based on Daly (1994).

Micromelerpeton credneri

- Character 61 (occiput [posterior margin]): 0 → 0&1: this is an updated score accounting for polymorphism documented by Boy (1995).
- Character 95 (humerus [supinator process]): 1 → 0: this is a correction.

Dendrysekos helogenes

- Character 26 (nasals [anterior extent]): 0 → 0&1: this is an updated score accounting for polymorphism documented by Holmes et al. (1998).
- Character 85 (prearticular): 1 → 0: this is a revised score informed by a closer examination of the range of prearticular morphology across dissorophids in which some taxa (e.g., *Anakamacops*) have a medial inflection more similar to trematopids compared to that of *Dendrysekos*.
- Character 99 (interclavicle): 0 → 1: this score is corrected; this is separate from the revised order of character states but was updated as a part of a reexamination of scores for this character.

Eryops megacephalus

- Character 21 (lacrimal-orbit): 1 → -: this is an updated score that reflects the partial dependency of any taxon with state 30-0 (prefrontal and jugal contact), as this sutural relationship will always result in the exclusion of the lacrimal from the orbit.
- Character 36 (postorbital): ? → 0: this is an updated score.
- Character 47 (semilunar flange): ? → 0: this is an updated score.
- Character 94 (cleithrum [head]): ? → 0: this is an updated score.

Proterogyrinus scheelei

- Character 6 (skull table-cheek): 1 → ?: this is a correction.
- Character 13 (septomaxilla): 0 → ?: this is a correction.

The following characters were removed (numbering from Gee, 2020):

- **Character 5 (snout shape)**: as listed by Schoch (2012), state 1 (pointed snout) is shared by *Dissorophus* and *Broiliellus brevis*. In previous matrices, state 1 has also been scored for *Broiliellus olsoni* and *Broiliellus texensis* and as polymorphic for *Conjunctio multidens*. The distinction between a "parabolic to square-shaped" snout and a "pointed" snout is not clear from figures, including those of Schoch (2012:fig. 1). *Broiliellus olsoni* actually appears closer to *Aspidosaurus binasser*, which has a very clearly squared-off snout, while Schoch's reconstruction of *Platyhystrix rugosa* is the only taxon with a clearly defined "point" at the end of the snout but is scored for state 0. Meanwhile, the distinction between taxa with more parabolic snouts that are scored as such (e.g., *Cacops morrisi*) and those purported to have a pointed snout is not apparent. The character is explicitly not about snout width, so I consider this character to be too arbitrarily defined to be useful at present.
- **Character 10 (narial elongation)**: this may seem a peculiar character to remove as it the elongate naris (10-1) is a trematopid synapomorphy. However, a non-elongate naris (10-0) was redundant with character states of at least two other characters (numbering from this study): 28-0 (prefrontal well separated from a non-elongate naris) and character 20-0 (lacrimal excluded from naris). If all taxa with state 10-0 were instead scored as inapplicable to remove the redundancy, the character would become parsimony-uninformative as a binary character, so it was therefore removed. The multiple states of characters 20 and 28 either implicitly or explicitly capture the narial elongation.
- **Character 22 (lacrimal-naris):** this character was removed in the same vein as the narial elongation character (partial redundancy), with one of its two states (lacrimal does not contact the naris) added as state 0 to the following multistate character (lacrimal-narial contribution). The resultant single character represents a more complete morphocline.
- **Character 31 (palpebral ossifications)**: although I have already taken a conservative approach to scoring this character, scoring it only when another form of scleral ossification was present as one way to assess whether the taphonomic conditions favored preservation of the loose palpebral ossifications, I am of the opinion that the character is generally not of much use for resolving the in-group relationships (that is to say that probably most if not all dissorophids, and terrestrial dissorophoids more broadly, had palpebral ossifications), and that including it with limited scores, in some cases based on a single specimen, might be more of a confounding or misleading datapoint. For example, not all skulls of *Doleserpeton annectens* preserve a palpebral ossification, even though the preservational conditions at Richards Spur favor the preservation of small loose skeletal elements. Similarly, I am somewhat skeptical that the micropholid *Pasawioops mayi* genuinely lacked these ossifications while the micropholid *Tersomius texensis* possessed a very well-developed palpebral cup. Fröbisch & Reisz (2012:appendix 2) similarly excluded this character on the same grounds.
- **Character 84 (ectopterygoid)**: this character was previously parsimony-uninformative and remained so in the updated version of my matrix, with only

Doleserpeton annectens (in which the presence of this bone is equivocal) scored for state 1.

The revised scoring of *Acheloma cumminsi* (inclusive of data from *Acheloma dunni*) that was used in Analysis 6 in the reanalysis of my old matrix (essentially testing whether inclusion of *Acheloma dunni* as a separate OTU and *Phonerpeton whitei* were confounding resolution) is listed here:

100200111011100[0 1]01003111110101100[0 1]01111110021000100001110000011[0 1]110100000010101001[0 1]0100

Note that brackets indicate polymorphism in TNT format and would need to be changed to parentheses in NEXUS format.

APPENDIX 3. Additional comments on character coding

The following appendix provides additional comments regarding the philosophy of how some nuanced characters were coded in this analysis and the philosophy of scoring features when they are only known in presumably immature individuals of a taxon. I refer the reader to Appendix S2 in Gee (2020) for comments on characters that were carried over from my previous analysis, as the utilized approaches were the same in this study. Comments on new characters are listed below:

- **Character 26 (LEE):** As with the preceding character (LEP), scoring accounted for how recently this exposure has been identified (Reisz, Anderson & Schoch, 2009). Unlike the LEP, which often forms a distinctly concave surface (e.g., Bolt, 1974b), the LEE is not as easily identified based on surficial topography alone. The observation that it is ontogenetically transient in at least *Cacops morrisi* also suggests that it can be quite small and therefore can only be reliably identified with good preservation in this region.
- **Character 66 (opisthotic-prootic):** I scored taxa for this character, even if both ossifications were not identified, as long as one ossification was preserved and appeared to be distinct and undamaged (e.g., *Platyhystrix rugosa*; Berman, Reisz & Fracasso, 1981).

Additionally, I scored cells as inapplicable (-) when the character was truly inapplicable, rather than as unknown (?) as is sometimes done by other workers since most programs cannot differentiate between these states (see Brazeau, Guillerme & Smith, 2017, for an algorithm that can, but Marjanović & Laurin, 2019:21, for critique of this approach). Therefore, any state scored as unknown is considered explicitly as such.

Dependent characters. Characters where one state was dependent on that of another character were also scored as inapplicable. On example is character 21 (lacrimal-orbit) and character 31 (prefrontal-jugal). The lacrimal may either enter (21-0) or be excluded from (21-1) the orbit when there is no prefrontal-jugal contact (30-1). However, when the prefrontal and the jugal meet (30-0), the lacrimal will always be excluded from the orbit (21-1). Therefore, for the two taxa with state 30-0 (*Eryops megacephalus* and *Chenoprosopus milleri*), character 21 is scored as inapplicable even though it is not truly inapplicable when viewed as a standalone character.

Reconciling character scoring with ontogenetic gaps. One other area that merits detailed discussion is how to score a character which can only be assessed in specimens that are relatively small for the taxon's known range (i.e. probable "juveniles" or "sub-adults"). This was not a frequent issue for trematopids because the taxa with wide size ranges were known from at least one large specimen that was usually subequal in informativeness to a smaller one (*Acheloma cumminsi*, *Mattauschia laticeps*). This was however a more recurring issue for dissorophids. For example, the palate of *Cacops woehri* is mostly known only from the holotype, which is about two-thirds the length of the largest known skulls. The conservative approach would be to only score from the largest known specimens, with an arbitrary cutoff for subequally sized specimens (e.g., within 10% of the size of the largest known specimen). In this example, the holotype would not be used, and *C. woehri* would be left unscored for most palatal characters. This approach is unattractive because it minimizes the amount of usable data.

The inverse approach would be to score any character that can be observed in any specimen regardless of inferred relative maturity. This is the most liberal approach and is often implicitly utilized to maximize the available data. In some instances, it may substantially expand the scoreable proportion of characters from the previous approach. However, it runs the risk of creating a chimeric set of scores that collectively represent an anatomy that would never be found in a single individual, which could then be misinterpreted as a taxon with a unique mixture of features.

The third approach is to score characters that can only be scored from small specimens when the feature or a specific condition appear unlikely to change ontogenetically. For example, there is no evidence or reason to assume that osteoderms were lost throughout ontogeny in any dissorophid. Therefore, "sub-adult" specimens of *Cacops morrisi* (OMNH 73206a, OMNH 79339), the only specimens of the taxon with substantial postcrania (Gee & Reisz, 2018a; Gee, Bevitt & Reisz, 2019) can be used to score the presence/absence of osteoderms. Other examples for *C. morrisi* are the position of the carotid arteries and the dorsal covering of the scapula by the cleithrum. This ontogeny-informed approach represents a balanced approach to maximizing data while avoiding chimeric OTUs, and this is the one that I use here. Features to exercise caution with are those that are gradational (e.g., tooth count, ratio-based characters) rather than binary characters; ontogenetically influenced characters where only the plesiomorphic (but possibly juvenile) condition is observed among known specimens (e.g., open basicranial articulation); and postcrania, for which ontogeny is poorly constrained (e.g., uncinate processes).

The status of *Kamacops acervalis*. As mentioned in the main text, there are a number of concerns with how this taxon has been previously scored. Firstly, the completeness of the material is unclear because previous figures have either consisted of close-up views of the occiput and parts of the braincase or of full cranial reconstructions; the only published photograph of any specimen is a snout (Schoch & Milner, 2014:fig. 37E). Although this snout is listed with the number of the holotype (PIN 3817/1), Gubin (1980), Schoch (1999), and Schoch & Milner indicate that the holotype is a skull lacking the preorbital region. Therefore, the photographed specimen is likely PIN 1758/332; regardless, the photograph and descriptions suggest that skull roof sutures are entirely unknown. This is reaffirmed by Schoch's (2012) reconstruction of the skull in dorsal view (figure 6 therein), which shows no sutures.

Additionally, the holotype and the referred material (three specimens) are not from the same sites. PIN 1758/332 and PIN 1758/333 (suggested to be osteoderms found next to 1758/332; Gubin, 1980) appear to have minimal if any skeletal overlap with the holotype. Gubin only briefly mentioned these specimens, and his rationale for their referral is unclear. The spatial separation and lack of skeletal overlap of specimens of *Kamacops acervalis* raises questions about their conspecificity, especially considering that there are other large dissorophids (*Iratusaurus*, *Zygosaurus*) from the same interval in Russia. Another specimen, originally identified as *Zygosaurus* but placed in *K. acervalis* by Gubin (1987), GCLU 1/1329, is not from the same province. While it preserves a large region of the mid-length of the skull that would overlap with at least PIN 1758/332, Gubin indicates that most of the specimen is an internal mold with minimal ornamentation preserved. Gubin did justify the referral, two features of which were carried forward to Schoch & Milner's diagnosis (tooth row between vomerine and palatine 'fangs' and flatter skull than *Zygosaurus*). Nonetheless, he also noted a prefrontal-postfrontal contact, a plesiomorphy unknown in any dissorophid. Because he indicated a 'cf.' designation for this specimen, reflecting his tentative placement, I did not consider it in scoring

K. acervalis (referred specimens of other taxa with a 'cf.' designation were not factored into species-level OTUs).

The ambiguity surrounding *Kamacops acervalis* is relevant in a few ways. The first is that there are no published data that would support scoring any characters related to cranial sutures, yet the taxon has been scored for numerous such characters in previous matrices (Schoch, 2012, and derivations thereof). Schoch did not indicate that he used GCLU 1/1329, and he scored the taxon as lacking a prefrontal-postfrontal contact, contrary to the specimen. The palate has only been figured in reconstructions (Gubin, 1980; Schoch, 1999). The second is the fidelity of these reconstructions; even if reconstructions are considered viable for scoring taxa, it is unclear whether enough demonstrably conspecific specimens are known to produce a full reconstruction. This in turn queries whether any characters involving skull length can be scored, including two of the three characters in which *Kamacops acervalis* differs from *Cacops* in previous matrices (distance from squamosal embayment to orbit; suborbital bar height).

APPENDIX 4. Revised character matrix of Gee (2020)

Please refer to the online supplemental information for the associated character matrix (.nex).

APPENDIX 5. Updated scorings to Dilkes (2020)

This appendix summarizes the scoring changes made to the matrix of Dilkes (2020), a derivation of Schoch (2012), that were used in the analysis of Analysis 7. The majority of scoring changes (65 of 129) are from cells that were previously scored but that can be determined to not be scoreable with a high degree of confidence. For example, several postcranial characters are scored for *Cacops woehri*, but this taxon is not known from any postcrania, including any unpublished material that I am aware of from the Richards Spur locality. There is obviously the possibility that unpublished material exists for some of the taxa whose scores I have modified, but it would not have been recently collected or prepared based on when most taxa were collected, and Schoch & Milner (2014) were thorough in listing specimens, including of unpublished material.

Therefore, these changes are regarded as unequivocal errors and number 73 in total. A minority of changes are from previously unscored characters that can be scored (35 of 143). Essentially none of these changes are based on recently published studies – in other words, the changes are not "updates" based on the expanding body of literature but are based on data that were overlooked in previous studies. For example, all of the new scores for *Fedexia striegeli* are based only on the original (and only) description by Berman et al. (2010). The remaining 35 changes are from one character state to another (or the introduction of a polymorphism).

If the scoring approach is "time-calibrated" in the sense of what was justified by the literature at the time of the original matrix's conception in 2012, this would result in a substantial amount of additional scored cells being changed to "?", especially for the three species of *Cacops*, whose osteology has been substantially expanded in recent years. Note that I do not adjust for scorings that are technically inapplicable (e.g., osteoderms in most trematopids) and that are scored as "-" in some matrices, including my own but that are scored as "?" since most phylogenetics algorithms cannot differentiate these states.

Most scores have been propagated essentially (or entirely) unchanged from Schoch (2012) to the most recent derivation of Dilkes (2020), excepting the scoring of new characters. A few taxa whose scores have been propagated forward were originally scored by other workers: *Broiliellus reiszi* (Holmes, Berman & Anderson, 2013); *Cacops woehri* (Holmes, Berman & Anderson, 2013); *Anakamacops petrolicus* (Liu, 2018); composite of *Conjunctio multidens* (Liu, 2018); *Reiszerpeton renascentis* (Liu, 2018); and *Diploseira angusta* (Dilkes, 2020). While a different composite of *Co. multidens* (Schoch & Sues, 2013), a novel scoring of *Ca. woehri* (Schoch & Sues, 2013), and the novel scoring of *R. renascentis* (Maddin et al., 2013) were produced by other workers, these scores were not provided in the publication or associated supplemental data, and these scores were not propagated by other workers.

Where appropriate, specific citations have been provided to justify the correction, otherwise the relevant literature is cited with the species. Taxa are listed in the order in which they appear in Dilkes' matrix.

Platyrhinops lyelli (sources: Clack & Milner, 2010)

1. Character 24 (palpebral ossicles): ? → 1. Clack & Milner (2010:289, figs. 1–2) report these ossifications.
2. Character 29 (septomaxilla): 0 → ?. Clack & Milner (2010:279) state that no septomaxilla is present in any specimen.

3. Character 45 (vomerine medium septum): 0 → ? Clack & Milner (2009) make no mention of a vomerine septum, and given the preservation of specimens of *P. lyelli*, it would be impossible to assess whether this ascending lamina was present.

Doleserpeton annectens (sources: Bolt, 1969; Sigurdsen & Bolt, 2010)

4. Character 32 (postorbital): 1 → 0. Both specimen illustrations and reconstructions show a triangular postorbital that tapers to a point (Sigurdsen & Bolt, 2010:figs. 2A, 4A).

Acheloma dunni (sources: Polley & Reisz, 2011)

5. Character 34 (vomerine ridge): 1 → 0. Polley & Reisz (2011:802) indicate the presence of a dentulous ridge medial to the choana, and this is an autapomorphy of *Acheloma* (Schoch & Milner, 2014; Gee, 2020).
6. Character 47 (stapedial foramen): 1 → 0. Polley & Reisz (2011:805) indicate the presence of a stapedial foramen.

- Character 68 (interclavicle): unchanged. This character is noted because the interclavicle is unknown in *Acheloma dunni* (sensu Polley & Reisz, 2011) and is an error if the synonymy with *A. cumminsi* (Gee, 2020) is not accepted. If the OTU is changed to the new *Acheloma cumminsi* (sensu Gee, 2020), then the feature is known based on material from Texas (Williston, 1909).

Phonerpeton pricei (sources: Olson, 1941; Dilkes, 1990, 1993)

7. Character 41 (internarial fenestra): 1 → 0&1. The holotype appears to lack a fenestra (Dilkes, 1990:225, fig. 2), as may one referred specimen (AMNH FARB 7150; Dilkes, 1990:fig. 3), but other referred specimens possess one (Dilkes, 1990:fig. 4A), and Dilkes reconstructed this taxon as having a fenestra.
8. Character 47 (stapedial foramen): 1 → ?. Dilkes (1990:233) makes brief mention of a stapes that is figured only in a low-brightness photograph (his fig. 2) in which the stapes is not discernible. While Dilkes did not mention a stapedial foramen, neither did he explicitly mention that one was absent. Figures by Olson (1941; as "*Acheloma pricei*") are equally uninformative.
9. Character 56 (postorbital-supratemporal): 0 → 0&1. This is a variable condition within AMNH FARB 7150 (Dilkes, 1990:fig. 3).
10. Character 61 (ilium): 1 → ?. No ilium is known for this taxon.

Anconastes vesperus (sources: Berman, Reisz & Eberth, 1987)

11. Character 4 (dorsal quadrate process): 1 → ?. Berman, Reisz & Eberth (1987:257) explicitly state that "it cannot be determined whether the otic notch was closed posteriorly by a posterodorsal process of the quadrate." Although they speculate that one may have been present, this score was changed here.
12. Character 11 (vomer-pterygoid): ? → 0. The vomer's posterior process clearly contacts the pterygoid, as seen through the orbits (Berman, Reisz & Eberth, 1987:fig. 7).
13. Character 17 (tabular size): 0 → ?. Berman, Reisz & Eberth (1987:258) indicate that the tabulars are incomplete; their estimation that they were of "moderate size" does not permit a scoring of this character alone.
14. Character 18 (tabular-squamosal): 1 → ?. This score should either be "?" or "0," but definitely not state 1. The figure of the right side of the skull (Berman, Reisz & Eberth, 1987:fig. 7) depicts a wide separation of these elements, but the tabular is also

clearly incomplete, missing all of the lateral margin, so I left it as unknown to be conservative, as I did in my own matrix.

15. Character 22 (prefrontal-postfrontal): 1 → ?. The lateral margin of the frontals and the medial margin of the orbits are clearly not preserved (Berman, Reisz & Eberth, 1987:fig. 7).
16. Character 23 (interorbital width): 0 → ?. As with character 22.
17. Character 32 (postorbital): 1 → 0. At least the left postorbital tapers to an acute point (Berman, Reisz & Eberth, 1987:fig. 7). The right postorbital is ambiguous because the posterior margin is partially squared-off, but a portion of it projects more posteriorly as a narrow process. The character should either be scored for state 0 or as polymorphic.
18. Character 38 (exoccipital-postparietal): ? → 0. Berman, Reisz & Eberth (1987:259) state that "their [the exoccipitals'] dorsal surfaces contact the occipital flanges of the postparietals."
19. Character 46 (tabular process): 0 → ?. As with character 17; both tabulars are incomplete posterolaterally, which is where the process would be developed.
20. Character 47 (stapedial foramen): 1 → 0. Berman, Reisz & Eberth (1987:260) indicate the presence of a stapedial foramen.
21. Character 64 (tabular horn): 0 → ?. As with characters 17 and 46.

Fedexia striegeli (sources: Berman et al., 2010)

22. Character 6 (parasphenoid dentition): ? → 0. Berman et al. (2010:309) indicate the presence of denticles on the basal plate.
23. Character 7 (parasphenoid denticle field): ? → 0. As with character 6.
24. Character 9 (vomerine denticle field): ? → 0. Berman et al. (2010:308) indicate the entire palate is covered in denticles.
25. Character 10 (vomerine fangs): ? → 0. The left vomer is sufficiently exposed to exclude the presence of additional 'fangs' (Berman et al., 2010:fig. 9).
26. Character 11 (vomer-pterygoid): ? → 0. The contact is clearly figured by Berman et al. (2010:fig. 9).
27. Character 15 (narial flange): ? → 1. The left naris is partially exposed to indicate the presence of a narial flange, which was described (Berman et al., 2010:304, fig. 5).
28. Character 24 (palpebral ossifications): 0 → 1. There is a large palpebral bone in the left orbit (Berman et al., 2010:fig. 5).
29. Character 32 (postorbital): 1 → 0. At least the right postorbital definitively tapers to a point (Berman et al., 2010:fig. 4). The left postorbital tapers but with a slightly rounded end. The character should either be scored for state 0 or as polymorphic.
30. Character 33 (vomerine process): ? → 0. Compared to other trematopids, the vomer clearly ends much more bluntly and without a slender, tapering process (Berman et al., 2010:fig. 9).
31. Character 35 (palatine-vacuity): ? → 0. In dissorophoids where there is a vomer-pterygoid contact (character 11), the palatine is always excluded from the interpterygoid vacuity.
32. Character 38 (exoccipital-postparietal): ? → 0. The contact is clearly shown by Berman et al. (2010:fig. 8).
33. Character 42 (marginal teeth): 0 → 1. The teeth are caniniform like those of other trematopids (Berman et al., 2010:306, figs. 5–6).

34. Character 43 (prearticular): 0 → ?. Only the anteriormost region of the lower jaws is preserved, and the matrix that may have infilled the rest of the jaw has been partially prepared, so its contour is not reliable for estimating the original bone (Berman et al., 2010:figs. 5–6, 8–9).
35. Character 45 (median vomerine septum): 0 → 1. Berman et al. (2010:308) state that the vomers are "moderately vaulted dorsally along the anterior portion of their midline union"; this appears to meet the criterion for a vomerine septum.
36. Character 47 (stapedial foramen): ? → 0. Berman et al. (2010:310) indicate the presence of a stapedial foramen.
37. Character 50 (squamosal): 0 → 1. Berman et al. (2010:306) describe a semilunar curvature.
38. Character 63 (carotid artery): 0 → ?. Berman et al. (2010:309) expressly state that "there is no evidence of foramina for the internal carotid arteries."

Tambachia trogallas (sources: Sumida et al., 1998)

39. Character 4 (dorsal quadrate process): 1 → ?. Sumida et al. (1998:617) state that the "only preserved and visible portion of the quadrates is the ventral surface of the left condyle…it is not possible to determine whether a posterodorsal process of the quadrate was present."
40. Character 25 (stapes): 1 → ?. There is no stapes described for this taxon.
41. Character 30 (parietal width): 1 → ?. Almost all of the parietals is lost; only the posterolateral regions are preserved, and their anterior extent is entirely unclear, thereby precluding a length estimate except from the reconstruction (Sumida et al., 1998:fig. 3).
42. Character 37 (exoccipital-tabular): 0 → ?. The exoccipitals are not preserved.
43. Character 39 (jaw articulation): 0 → ?. As with character 37.
44. Character 47 (stapedial foramen): 1 → ?. As with character 25.

Ecolsonia cutlerensis (sources: Berman, Reisz & Eberth, 1985)

45. Character 38 (exoccipital-postparietal): ? → 0. Berman, Reisz & Eberth (1985:16) identify this contact.
46. Character 47 (stapedial foramen): 1 → ?. Berman, Reisz & Eberth (1985:16) tentatively identified a stapes in the right otic notch of one specimen. While the interpretation seems correct, the stapes would then be in articulation and the footplate where the stapedial foramen occurs is obscured. There is only one sentence describing the stapes, and neither that nor the figure indicate a stapedial foramen.

Aspidosaurus binasser (sources: Berman & Lucas, 2003)

47. Character 11 (vomer-pterygoid): 1 → ?. Berman & Lucas (2003:250) state that the palatal sutures are not traceable other than the ectopterygoid-pterygoid separation.
48. Character 17 (tabular size): 0 → ?. Berman & Lucas (2003:249) state that the postparietal and the tabular are incomplete. As preserved, the tabular is much narrower and smaller than the postparietal, so the character should either be scored for state 1 or as unknown.
49. Character 23 (interorbital distance): 0 → ?. The interorbital region is not preserved in the holotype (Berman & Lucas, 2003:fig. 2). Furthermore, no other character based on complete skull length (not confidently known) is scored.
50. Character 33 (vomerine process): 0 → ?. As with character 11.

51. Character 38 (exoccipital-postparietal): ? → 0. Berman & Lucas (2003:251) state that these elements contact.
52. Character 41 (internarial fenestra): ? → 1. Berman & Lucas (2003:248-249, fig. 3) identify this opening.
53. Character 44 (otic notch): 0 → ?. Berman & Lucas (2003:249) indicate that only the dorsal portion of the otic notch is present.
54. Character 51 (dorsal rim of occiput): ? → 0. Berman & Lucas (2003:249) indicate there is no nuchal ridge.
55. Character 65 (jugal ornamentation): 0 → ?. The jugal is only preserved anteriorly (Berman & Lucas, 2003:fig. 2), whereas taxa with an ornamented jugal have the structure on the posterior region.

Platyhystrix rugosa (sources: Langston, 1953; Berman, Reisz & Fracasso, 1981)

56. Character 38 (exoccipital-postparietal): ? → 0. Berman, Reisz & Fracasso (1981:406) identify this contact.
57. Character 39 (jaw articulation): 0 → ?. The quadrate is entirely unknown.

Broiliellus texensis (sources: Williston, 1914)

58. Character 9 (vomerine denticle field): ? → 0. Williston (1914:53) indicates the entire vomer is covered in denticles.
59. Character 24 (palpebral ossifications): ? → 1. Williston (1914:50) identifies palpebral ossifications.

Broiliellus brevis (sources: Carroll, 1964a)

60. Character 32 (postorbital): 0 → 1. Neither postorbital tapers to an acute point, although both taper in width, and the right postorbital is a square element with one corner directed posteriorly (Carroll, 1964a:fig. 9A). Based on how other taxa are scored, this taxon should not be scored for state 0.
61. Character 59 (osteoderm width): ? → 0. Carroll (1964a:197-198) indicates armor that is much narrower than that of *B. texensis*.
62. Character 9 (vomerine denticle field): 1 → 0. Carroll (1964a:197, fig. 10A) illustrates the vomers as being denticulate like the other palatal bones, and there is no explicit rationale there or anywhere else for identifying the holotype as a distinct juvenile.
63. Character 27 (maxilla dentition): ? → 0. Carroll (1964a:fig. 9B) illustrate the teeth as extending well back of the orbit.
64. Character 29 (septomaxilla): 1 → ?. No septomaxilla is illustrated or described.
65. Character 32 (postorbital): ? → 0. The postorbital is complete on both sides (Carroll, 1964a:fig. 9A).

Broiliellus olsoni (sources: DeMar, 1967)

66. Character 17 (tabular size): 0→ ?. DeMar (1967:126) indicates the tabular is not preserved.
67. Character 18 (tabular-squamosal): 1 → 0. As with character 17.
68. Character 30 (parietal width): 1 → ?. There is no indication that any of the parietals are complete posteriorly.
69. Character 32 (postorbital): 0 → 1. The left postorbital has a straight horizontal posterior margin (DeMar, 1967:fig. 2).
70. Character 39 (jaw articulation): 2 → ?. Neither the exoccipitals nor the quadrates are preserved in the holotype (DeMar, 1967:fig. 2), and there is no mention of the

exoccipitals for the referred specimen with the quadrate preserved. Other characters related to the exoccipitals were not previously scored.

71. Character 46 (tabular process): 0 → ?. As with characters 17 and 18.
72. Character 50 (squamosal): ? → 1. DeMar (1967:126, fig. 2B) depicts and describes a semilunar curvature.
73. Character 52 (preorbital-postorbital ratio): 1 → ?. No skull is complete (DeMar, 1967:123).
74. Character 53 (suborbital bar height): 1 → ?. As with character 52.
75. Character 54 (otic notch-orbit): 1 → ?. As with character 52.

Brevidorsum profundum (sources: Carroll, 1964a)

76. Character 32 (postorbital): 0 → 1. Neither postorbital tapers to an account point (Carroll, 1964a:fig. 15).
77. Character 40 (external naris): 0 → ?. No part of the external naris is preserved (Carroll, 1964a:fig. 15).
78. Character 53 (suborbital bar height): 1 → ?. The skull is clearly incomplete longitudinally, and the preceding character for preorbital-postorbital ratio is not scored.
79. Character 54 (otic notch-orbit): 1 → ?. As with character 53.

Conjunctio multidens (sources: Case & Williston, 1913; Carroll, 1964a; Schoch & Sues, 2013)

80. Character 67 (pointed snout): 0&1 → 0. As originally scored by Schoch (2012), both the holotype and the "Rio Arriba Taxon" (UCMP 40103) lack this feature. However, it is scored for the derived state for the "Admiral Taxon," which is one line down from the "Rio Arriba Taxon." Holmes et al. inverted the scores between these taxa for this character, which seems to be an error as a result of the original matrix not being provided in an accessible format in the publication (a figure of text strings) because they did not mention this scoring change but did mention other scoring changes. This typographic error then resulted in a polymorphism in Liu's (2018) composite scoring of *C. multidens*, which was carried forward.

Scapanops neglectus (sources: Carroll, 1964a; Schoch & Sues, 2013)

81. Character 24 (palpebral ossifications): ? → 1. Schoch & Sues (2013:441, fig. 1A-B) identify these features.
82. Character 25 (stapes): 1 → ?. There is no stapes described or figured by Carroll (1964a) or Schoch & Sues (2013), and the loss of the palate and preparation of matrix to reach the ventral surface of the skull roof in some areas indicates that none is present.
83. Character 39 (jaw articulation): 2 → ?. Neither the exoccipitals nor the quadrate is preserved.
84. Character 48 (stapedial foramen): 0 → ?. As with character 25.
85. Character 62 (parasphenoidal plate): 0 → ?. The palate is not preserved (Schoch & Sues, 2013:441).
86. Character 67 (pointed snout): 0 → 1. Following the same character for *Conjunctio multidens* and the original scoring of Schoch (2012) that seems to have been accidentally changed by Holmes, Berman & Anderson (2013).

Cacops morrisi (sources: Reisz, Schoch & Anderson, 2009; Gee & Reisz, 2018a; Gee, Bevitt & Reisz, 2019)

87. Character 27 (maxilla dentition): 1 → 0. The tooth row extends slightly posterior to the orbit (Reisz, Schoch & Anderson, 2009; Gee & Reisz, 2018a:6, figs. 3–4, 6).
88. Character 34 (vomerine tooth row): 0 → 1. Schoch has consistently reconstructed this taxon as having a row of sizeable teeth only slightly smaller in circumference than the marginal teeth in a position along the medial choanal margin (e.g., Schoch, 2012:fig. 3B), but this seems to be a misinterpretation of the overly simplified reconstructions of Reisz, Schoch & Anderson (2009:fig. 3) in which there are denticles indicated in this position but nowhere else on the palate. However, Reisz, Schoch & Anderson clearly note that small teeth cover "most of the palatal surfaces," and therefore the restricted depiction of non-'tusk/fang' palatal dentition to this margin is an oversimplification that inadvertently suggests that these teeth are larger than those on the rest of the palate. Specimen illustrations (Gee & Reisz, 2018a:figs. 3–4) clearly show that not to be the case.
89. Character 38 (exoccipital-postparietals): 1 → 0. The separation of these elements has been cited as a cacopine feature (e.g., Schoch, 2012), but Reisz, Schoch & Anderson (2009:5) explicitly state that "the postparietals further bear paired posterolateral projections (occipital flanges) that were sutured to the vertical columns of the exoccipital."
90. Character 43 (prearticular): 0 → 1. the small, referred specimen (OMNH 53077) more fully figured by Gee & Reisz (2018a:fig. 4) shows a clear medial inflection.
91. Character 50 (squamosal): 1 → 0. while the small OMNH 53077 has a semilunar curvature, all other specimens do not, indicating an ontogenetically influenced feature that should only be scored based on the larger specimens (Reisz, Schoch & Anderson, 2009; Gee & Reisz, 2018a:figs. 3–4, 6).
92. Character 61 (ilium): 1 → ?. The ilium is unknown for this taxon. While Gee & Reisz (2018b) refer a well-exposed pelvis to cf. *Cacops*, that designation is provisional and cannot be assigned to a particular species of *Cacops*.

Cacops aspidephorus (sources: Williston, 1910; Anderson, 2005; Dilkes & Brown, 2007; Dilkes, 2009; Anderson, Scott & Reisz, 2020)

93. Character 6 (parasphenoid dentition): 1 → 0. Anderson, Scott & Reisz (2020:7) state that "what little surface [between the basal tubera and the basicranial articulations] is covered by a shagreen of denticles that does not appear to be on a raised patch."
94. Character 7 (parasphenoid denticle field): ? → 0. Following character 6 and no indication of denticles on the cultriform process.
95. Character 9 (vomerine denticle field): ? → 0. There is a poorly preserved denticle field at least on the posterior half of a referred specimen (UMMP 3417), and Anderson, Scott & Reisz (2020:8) state that denticles are found on most of the palatal surfaces.
96. Character 11 (pterygoid-vomer): 1 → ?. The pterygoid is incomplete anteriorly, and the vomer posteriorly, in the only specimen with defined palatal sutures (UMMP 3417; Anderson, Scott & Reisz:fig. 2).
97. Character 18 (tabular-squamosal): 1 → 0. The elements are widely separated in the otic notch of FMNH UC 900 in which the smooth flange is well-exposed (Anderson, Scott & Reisz (2020:fig. 3).
98. Character 27 (maxilla dentition): 1 → 0. Figures of UMMP 3417 indicate the teeth end just slightly posterior to the orbit (Anderson, Scott & Reisz, 2020:fig. 2).

99. Character 34 (vomerine tooth row): ? → 1. Anderson, Scott & Reisz (2020:fig. 2) clearly show a lack of any tooth row.
100. Character 33 (vomerine process): 0 → ?. The vomer is too incomplete to discern.
101. Character 38 (exoccipital-postparietal): 1 → 0. The sutures are not entirely resolved, but Anderson et al. (2020:7) indicate a "trace of a suture between the exoccipitals and the occipital laminae of the postparietals."
102. Character 41 (internarial fenestra): 1 → ?. The complete but badly preserved holotype shows no evidence of a fenestra (Williston, 1910), and the midline of FMNH UC 900 is practically straight (Anderson, Scott & Reisz, 2020:fig. 3). The latter study suggested the possible presence of a fenestra based on UMMP 3417 (p. 6 therein), but they did not reconstruct the skull with the fenestra (fig. 9 therein). The more conservative correction in this instance is "?"
103. Character 56 (postorbital-supratemporal): ? → 1. This is clearly observed in UMMP 3417 (Anderson, Scott & Reisz, 2020:fig. 2).
104. Character 50 (squamosal): 1 → 0. semilunar curvature is not apparent from any specimen.

Cacops woehri (sources: Fröbisch & Reisz, 2012; Fröbisch, Brar & Reisz, 2015; Gee, Bevitt & Reisz, 2019)

105. Character 4 (dorsal quadrate process): 1 → ?. The quadrate is entirely unknown in this taxon. The isolated jaw articulation referred to cf. *Cacops woehri* by Gee, Bevitt & Reisz (2019:9–10, fig. 9.7–9.14) does not share any skeletal overlap with other diagnostic material and is not considered here.
106. Character 5 (vomerine depression): 1 → ?. The anteromedial region of the palate is unknown in this taxon; Fröbisch & Reisz (2012:40) give no indication of any depression and explicitly call the element "flat" and "sheet-like."
107. Character 9 (vomerine denticle field): ? → 0. There is a poorly preserved denticle field in the holotype (Fröbisch & Reisz, 2012:fig. 3), but a uniformly distributed field was confirmed in a referred specimen (ROMVP 80800) by Gee, Bevitt & Reisz (2019:fig. 8).
108. Character 15 (narial flange): 1 → ?. In all specimens with the nostril preserved (Fröbisch & Reisz, 2012:fig. 2; Gee, Bevitt & Reisz, 2019:figs. 7–8), it is infilled with matrix such that neither the medial surface nor the ventral surface is exposed; it is thus not possible to determine whether this flange was present.
109. Character 25 (stapes): 1 → ?. The stapes is unknown in this taxon.
110. Character 27 (maxilla dentition): 1 → 0. The tooth row extends slightly posterior to the orbit in the holotype (Fröbisch & Reisz, 2012:39).
111. Character 34 (vomerine tooth row): ? → 1. As with character 9.
112. Character 37 (exoccipital-tabular): 0 → ?. While Fröbisch & Reisz (2012:37) suggest that these elements contact, the exoccipitals are not preserved in the holotype or in any subsequently described specimen, and the authors' conjecture is clearly based on the condition seen in all other olsoniforms rather than even a tentatively identified fragment.
113. Character 39 (position of the jaw articulation): 0 → ?. The quadrates are entirely unknown in this taxon, as are the exoccipitals; as both of these are the landmarks prescribed by the character, it cannot be scored.
114. Character 47 (stapedial foramen): 0 → ?. As with character 25.

115. Character 48 (tubercular ornamentation): 1 → 0. The absence of tubercular ornamentation is an explicit differential feature of this species of *Cacops* (Fröbisch & Reisz, 2012:36; Gee & Reisz, 2018a:3).
116. Character 56 (postorbital-supratemporal): ? → 0. This contact is preserved in both the holotype (Fröbisch & Reisz, 2012:fig. 1) and the referred specimen described by Fröbisch, Brar & Reisz (2015:fig. 2).
117. Character 58 (osteoderms): 1 → ?. Postcrania are unknown for this taxon.
118. Character 59 (osteoderm width): 0 → ?. As with character 58.
119. Character 61 (ilium): 1 → ?. As with character 58.
120. Character 69 (supinator process): 1 → ?. As with character 58.
121. Character 70 (entepicondylar foramen): 1 → ?. As with character 58.

Kamacops acervalis (sources: Gubin, 1980; Schoch, 1999)

***Note:** for this taxon, cranial sutures have never been illustrated, reconstructed, or described (Gubin, 1980; Schoch 1999). If the specimen described by Gubin (1987) is accepted to be not only *Kamacops* (Gubin expressed some doubt) but *K. acervalis* specifically (additional doubt and a 'cf.' designation), some features could be scored, and at least one would have to be changed (prefrontal-postfrontal contact). Previous workers have not indicated that this specimen was incorporated into the scoring. Therefore, every feature listed here is changed from being scored to being left unscored because these characters require the sutures to be known, and Gubin's (1987) specimen is not utilized.

122. Character 16 (prefrontal process): 1 → ?.
123. Character 17 (tabular size): 0 → ?.
124. Character 18 (tabular-squamosal contact): 1 → ?.
125. Character 19 (squamosal-supratemporal suture): 1 → ?.
126. Character 22 (prefrontal-postfrontal): 1 → ?.
127. Character 26 (prefrontal-jugal): 0 → ?.
128. Character 30 (parietal width): 1 → ?.
129. Character 49 (lacrimal-naris): 0 → ?.
130. Character 50 (squamosal): 1 → ?.
131. Character 57 (intertemporal): 1 → ?.

Zygosaurus lucius (sources: Eichwald, 1848; Efremov, 1937)

132. Character 19 (supratemporal-squamosal): 1 → ?. Almost the entire skull roof is lost in the holotype (Eichwald, 1848:pl. 2.2), and these two elements are clearly not sufficiently complete for this character to be scored (if they are preserved at all).

Reiszerpeton renascentis (sources: Maddin et al., 2013)

133. Character 2 (LEP shape): 0 → 0&1. The LEP is barely exposed in lateral view on the left side and merely lies on top of the maxilla, whereas this exposure is broad and incises ventrally into the maxilla on the right side to create a prominently stepped dorsal margin of the maxilla. Neither side appears deformed in a way that alters the nature of the exposure.
134. Character 3 (maxilla contribution to orbit): 1 → 0. The maxilla appears to enter the orbit only on the right side of the skull, which has been crushed and folded under the skull (Maddin et al., 2013:454, fig. 5C). Close examination of the photographs shows that the orbital margin is not clearly continuous or smooth in the region of purported maxillary contact. The taxon should either be scored as polymorphic, or, as

I have done here and in my own matrix, for the unequivocal condition of the left side (absent).

135. Character 6 (parasphenoid dentition): 1 → ?. Maddin et al. (2013) gives no indication in the text of the distribution of palatal dentition other than the 'fangs,' and the figure should not be taken as a faithful representation of the biological condition since there were almost certainly denticles on some part of the palate, even if not on the parasphenoid.
136. Character 10 (vomerine fangs): ? → 0. Maddin et al. (2013:456, fig. 5B) were able to identify the intervomerine fossa / depression, which indicates enough of at least one vomer is present medially to rule out the presence of additional 'fangs' like in micropholids.
137. Character 53 (suborbital bar height): 0 → ?. Like other characters that rely on the full skull length to be known, which are left unscored in the original matrix, this character should not be scored since the reference point is unknown.
138. Character 54 (otic notch-orbit distance): 1 → ?. As with character 53.
139. Character 63 (carotid artery): ? → 1. This was the one feature cited by Maddin et al. (2013:457) for supporting *R. renascentis* as a cacopine.

Anakamacops petrolicus (sources: Li & Cheng, 1999; Liu, 2018)

140. Character 28 (skull outline): ? → 0. Following precedent of scoring based on all available specimens, the smaller holotype was incorporated based on other scored characters (e.g., 41 – internarial fontanelle) that can only be scored from this specimen. The holotype is therefore reliable for scoring this feature.
141. Character 29 (septomaxilla): 1 → 0. Neither the original description (Li & Cheng, 1999) nor the latest one (Liu, 2018) describe or figure a septomaxilla.
142. Character 53 (suborbital bar height): 0 → ?. Like other characters that rely on the full skull length to be known, which are left unscored in the original matrix, this character should not be scored since the reference point is unknown.
143. Character 38 (exoccipital-postparietal): 1 → 0. The sutures are not entirely resolved, but Liu (2018:6) explicitly states that "two exoccipitals suture with the postparietal."

APPENDIX 6. Revised character matrix of Dilkes (2020)

Please refer to the online supplemental information for the associated character matrix (.nex).

APPENDIX 7. Associated .tre files for all analyses

Please refer to the online supplemental information for the associated sets of MPTs (.tre).

APPENDIX 8. Summary of previous changes to Schoch's (2012) matrix

This appendix aggregates the published data available from previous publications regarding changes made to the original matrix of Schoch (2012) or a direct derivation thereof. This appendix does not contain less derived derivations such as Schoch (2018) or Gee et al. (2021) where a substantial number of characters come from other sources. Where unspecified, the character number refers to that of the matrix of the study under which it is described, not the original numbering of Schoch's.

Holmes, Berman & Anderson (2013)

- Source: Schoch (2012)
- Matrix available: Yes, as NEXUS text file
- Taxa added: *Broiliellus reiszi, Cacops woehri*
- Taxa modified: None
- Taxa removed: None
- Characters added:
  - Schoch's four-state character 1 (LEP) was split into binary characters 1 and 2. The new character 1 is restricted to a characterization of the LEP by presence or absence (states 0 and 1 of Schoch's character), and the new character 2 is restricted to the separation of the jugal and the lacrimal by the maxilla. The new character 2 is technically the reduced equivalent of Schoch's character 1, thereby shifting all 70 of Schoch's characters up by one in the numbering scheme (i.e. Schoch's #1 is #2 in this matrix and that of Liu, 2018)
- Characters deleted: None
- Characters modified:
  - Schoch's characters 17 and 30 were modified in language.
- Characters rescored in their entirety:
  - Character 30 of Schoch (31 of Holmes et al.)
- Individual scoring changes:
  - Rio Arriba Taxon, #24: ? → 1
  - Rio Arriba Taxon, #46: 0 → 1
  - *Broiliellus texensis*, #52: 1 → 2
  - Rio Arriba Taxon, #64: 0 → 1
- Comments:
  - Note that as described in the main text, it appears that there was a typographic error during the transcribing of Schoch's matrix for character 67 (#66 of Schoch, 2012) for the "Admiral Taxon" and *Conjunctio* (which referred only to the holotype at the time). This was not reported by Holmes, Berman & Anderson, but their supplemental matrix shows that the scores for these two taxa were inverted for this character, perhaps because they are successive lines and were thus misread.

Maddin, Fröbisch, Evans, & Milner (2013)

- Source: Schoch (2012)
- Matrix available: No

- Taxa added: *Reiszerpeton renascentis*
- Taxa modified: None
- Taxa removed: None
- Characters added: None
- Characters deleted: None
- Characters modified: None
- Characters rescored in their entirety: None
- Individual scoring changes: None

Schoch & Sues (2013)

- Source: Schoch (2012)
- Matrix available: No
- Taxa added: *Cacops woehri*
- Taxa modified:
    - New composite of *Conjunctio multidens* by merging with "Rio Arriba Taxon" OTU.
- Taxa removed: *Brevidorsum profundum*, *Broiliellus olsoni* (note that these authors erroneously claimed on p. 443 that they had excluded *Broiliellus texensis*)
- Characters added: None
- Characters deleted: None
- Characters modified: None
- Characters rescored in their entirety: None
- Individual scoring changes: None

Liu (2018)

- Source: Holmes et al. (2013)
- Matrix available: Yes, as TNT file
- Taxa added: *Anakamacops petrolicus, Reiszerpeton renascentis*
- Taxa modified:
    - New composite of *Conjunctio multidens* by merging with "Rio Arriba Taxon" OTU.
- Taxa removed: None
- Characters added: None
- Characters deleted: None
- Characters modified:
    - Character 49 (subnarial process of lacrimal): additional character state added to cover taxa in which there is no subnarial process.
    - Character 53 (suborbital bar height): character redefined as relative to orbit height rather than to total skull length.
    - Character 59 (osteoderm width): noted state 2 in text; previously scored but not mentioned in character list.
    - Character 68 (interclavicle): noted state 2 in text; previously scored but not mentioned in character list.
- Characters rescored in their entirety: None
- Individual scoring changes:

- *Kamacops acervalis*, #54: 2 → 1
- *Zygosaurus lucius*, #54: 2 → 1

Dilkes (2020)

- Source: Liu (2018)
- Matrix available: Yes, as NEXUS file
- Taxa added: *Diploseira angusta*
- Taxa modified: None
- Taxa removed: None
- Characters added:
  - Character 2 (shape of LEP)
  - Character 72
  - Character 73
  - Character 74
  - Character 75
  - Character 76
  - Character 77
- Characters deleted:
  - Character 18 (postparietal shape) from Holmes et al. (2013); this is character 17 from Schoch (2012) and has often been suggested to be redundant with character 31/30 (postparietal proportions).
- Characters modified:
  - Character 48 (tubercular ornamentation): character renamed from "knobby exostoses" of previous studies.
  - Character 49 (subnarial process of lacrimal): character states modified to cover taxa in which the lacrimal does not form the ventral narial margin; quantitative specifiers added to states 1 and 2.
  - Character 53 (suborbital bar height): reverted to original definition by relation to skull length.
  - Character 59 (osteoderm width): reduced to binary character ('narrow' vs. 'wide').
- Characters rescored in their entirety: None
- Individual scoring changes:
  - *Tambachia trogallas*, #1: 1 → 0
  - *Ecolsonia cutlerensis*, #1: 0 → 1
  - *Platyrhinops lyelli*, #1: 1 → ?
  - *Dissorophus multicinctus*, #8: ? → 0
  - *Dissorophus multicinctus*, #24: ? → 1
  - *Dissorophus multicinctus*, #27: ? → 1
  - *Dissorophus multicinctus*, #32: 0 → 0&1
  - *Dissorophus multicinctus*, #39: 2 → 1
  - *Dissorophus multicinctus*, #41: 0 → ?
  - *Dissorophus multicinctus*, #46: 0 → 2
  - *Dissorophus multicinctus*, #50: ? → 1
  - *Broiliellus texensis*, #65: 0 → 1

APPENDIX 9. Skull measurement data for olsoniforms

This appendix contains Supplemental Table 3, the skull length dataset used to produce Figure 26 in the main text, and some comments on how these measurements were compiled.

In my previous study (Gee, 2020), I compiled measurements from the literature and also estimated measurements for specimens that had not been provided previously. As there was a reduced focus on individual specimens and their intra- or interspecific size disparity here, and only an interest in demonstrating general size patterns (not the distribution of sizes for any given taxon), the dissorophid sample is less comprehensive. The trematopid data are carried over from Gee (2020). I then surveyed the literature for measurements of complete skulls as well as approximations of incomplete skulls for dissorophids. Where estimates for incomplete skulls were not available, I did not generate an estimate of my own unless there was a complete skull of the same taxon that was sufficient to be used as a reference. In general, relatively incomplete skulls were not included unless they differed appreciably from a more complete skull. Estimations from other specimens were made for some specimens of *Cacops morrisi* and *Cacops woehri*. Taxa represented only by incomplete specimens were usually not sampled as a result: *Aspidosaurus chiton*, *Aspidosaurus novomexicanus*, *Brevidorsum profundum*, *Broiliellus olsoni*, *Nooxobeia gracilis*, *Parioxys bolli*, and *Reiszerpeton renascentis* were not sampled. None of these taxa contradict the general patterns that I remarked on in the text (e.g., dissorophines tend to be smaller than other taxa) given the small size (inferred skull length <7.5 cm) of most of these taxa and the taxonomic uncertainties over the large *N. gracilis* and *P. bolli*. I did not include the holotype of "*Trematopsis seltini*" since Milner's (1985) provisional assignment to *Cacops aspidephorus* was made at a time when *Cacops* was thought to be monospecific, and the specimen does not preserve clear autapomorphies of this species (the genus-level referral seems justified, however). The same was applied to the provisional referred specimen of *Kamacops acervalis* described by Gubin (1987).

Supplemental Table 3.

Listing of specimens incorporated into Figure 26, comparing skull lengths known for olsoniform taxa. Taxon names reflect current taxonomy and thus do not differentiate specimens originally referred to junior synonyms (e.g., "*Acheloma dunni*," "*Longiscitula houghae*"). Length measurements are given in cm.

Taxon	Length	Specimen	Source
Acheloma cumminsi	15.5	AMNH FARB 4205	Dilkes & Reisz (1987)
Acheloma cumminsi	17.7	FMNH UC 640	Dilkes & Reisz (1987)
Acheloma cumminsi	17.2	CMNH 10969	Olson (1970)
Acheloma cumminsi	15.6	MU 501	Mehl (1926)
Acheloma cumminsi	7	FMNH UC 1584	Olson (1941)
Acheloma cumminsi	16.4	OMNH 73281	Polley & Reisz (2011)
Acheloma cumminsi	16.4	BMRP 2007.3.1	Polley & Reisz (2011)
Acheloma cumminsi	5.6	BMRP 2007.3.4	Polley & Reisz (2011)
Acheloma cumminsi	7.1	OMNH 73493	Maddin, Reisz & Anderson (2010); Gee (2020)

Acheloma cumminsi	12.3	OMNH 73494	Maddin, Reisz & Anderson (2010); Gee (2020)
Acheloma cumminsi	15.3	OMNH 73509	Maddin, Reisz & Anderson (2010); Gee (2020)
Acheloma cumminsi	15.3	OMNH 73511	Maddin, Reisz & Anderson (2010); Gee (2020)
cf. *Acheloma cumminsi*	7.6	OMNH 79318	Gee, Bevitt & Reisz (2019)
Actiobates peabodyi	5.9	KUVP 17941	Gee & Reisz (2020)
Anakamacops petrolicus	20	ICGAS V 365	Liu (2018)
Anakamacops petrolicus	40	IVPP V 23862	Liu (2018)
Anconastes vesperus	9.9	CM 41711	Berman, Reisz & Eberth (1987)
Aspidosaurus binasser	19.5	TMM 43531-1	Berman & Lucas (2003)
Broiliellus arroyoensis	9.6	FMNH UR 431	DeMar (1967)
Broiliellus brevis	5.9	MCZ 1424	Carroll (1964)
Broiliellus reiszi	9.6	CM 41705	Holmes, Berman & Anderson (2013)
Broiliellus texensis	8.45	FMNH UC 684	Williston (1914)
Broiliellus texensis	7.3	FMNH UC 685	Williston (1914)
Cacops aspidephorus	11.7	FMNH UC 647	Anderson, Scott & Reisz (2020)
Cacops aspidephorus	12	FMNH UC 649	Anderson, Scott & Reisz (2020)
Cacops aspidephorus	11.5	FMNH UC 900	Anderson, Scott & Reisz (2020)
Cacops morrisi	10.8	OMNH 53077	Reisz, Anderson & Schoch (2009)
Cacops morrisi	7.4	OMNH 53073	Reisz, Anderson & Schoch (2009)
Cacops morrisi	8.2	OMNH 73206a	Gee & Reisz (2018)
Cacops morrisi	7.5	OMNH 73206c	Gee & Reisz (2018)
Cacops morrisi	9.3	OMNH 73206c	Gee & Reisz (2018)
Cacops morrisi	8.5	OMNH 79339	Gee, Bevitt & Reisz (2019)
Cacops woehri	6.2	OMNH 73216	Fröbisch & Reisz (2012)
Cacops woehri	7.75	BMRP 2007.3.5	Fröbisch, Brar & Reisz (2015)
Cacops woehri	8.4	OMNH 79338	Gee, Bevitt & Reisz (2019)
Cacops woehri	7.37	OMNH 80800	Gee, Bevitt & Reisz (2019)
Cacops woehri	6.8	OMNH 80801	Gee, Bevitt & Reisz (2019)
Conjunctio multidens	11.3	FMNH 673	Schoch & Sues (2013)
Conjunctio multidens	6.1	UCMP 40103	Schoch & Sues (2013)
Conjunctio multidens	4.5	CM 91215	Gee et al. (2021)
Dissorophus multicinctus	12.5	FMNH UR 430	DeMar (1966)
Dissorophus multicinctus	13.7	MCZ 2122-1	DeMar (1968)
Dissorophus multicinctus	10.9	MCZ 2122-3	DeMar (1968)
Dissorophus multicinctus	12.3	AMNH FARB 4376	DeMar (1968)
Dissorophus multicinctus	13.2	FMNH UC 648	DeMar (1968)
Ecolsonia cutlerensis	13.5	UCLA VP 1734	Berman, Reisz & Eberth (1985)
Ecolsonia cutlerensis	13.5	CM 41703	Berman, Reisz & Eberth (1985)
Ecolsonia cutlerensis	13.5	CM 38017	Berman, Reisz & Eberth (1985)

Fedexia striegeli	10.3	CM 76867	Berman et al. (2010)
Iratusaurus vorax	25	Not specified	Schoch & Milner (2014)
Kamacops acervalis	24	Not specified	Schoch & Milner (2014)
Mattauschia laticeps	3.3	NMP M 470	Milner (2018)
Mattauschia laticeps	12	MNMW 1989-10-47	Milner (2018)
Mordex calliprepes	3	MNHUK VP R2817	Milner (2018)
Parioxys ferricolus	16	Not specified	Schoch & Milner (2014)
Phonerpeton pricei	6.2	MCZ 1419	Dilkes (1990)
Phonerpeton pricei	7.2	AMNH 7150	Dilkes (1990)
Phonerpeton pricei	6.8	MCZ 1414	Dilkes (1990)
Phonerpeton sp.	7.4	MCZ 2475	Dilkes (1993)
Phonerpeton whitei	5.3	MCZ 2531	Olson (1941)
Phonerpeton whitei	6	MCZ 1767	Olson (1941)
Platyhystrix rugosa	19	AMNH FARB 11545	Berman, Reisz & Fracasso (1981)
Rotaryus gothae	4.8	MNG 10182	Berman et al. (2011)
Scapanops neglectus	7.9	MCZ 2369	Schoch & Sues (2013)
Tambachia trogallas	6.5	MNG 7722	Sumida et al. (2011)
Zygosaurus lucius	17	Not specified	Schoch & Milner (2014)

References

Anderson JS, Henrici AC, Sumida SS, Martens T, Berman DS. 2008b. *Georgenthalia clavinasica*, a new genus and species of dissorophoid temnospondyl from the Early Permian of Germany, and the relationships of the family Amphibamidae. *Journal of Vertebrate Paleontology* 28:61–75. DOI: 10.1671/0272-4634(2008)28[61:GCANGA]2.0.CO;2

Anderson JS, Reisz RR, Scott D, Fröbisch NB, Sumida SS. 2008a. A stem batrachian from the Early Permian of Texas and the origin of frogs and salamanders. *Nature* 453:515–518. DOI: 10.1038/nature06865

Anderson JS, Scott D, Reisz RR. 2020. The anatomy of the dermatocranium and mandible of *Cacops aspidephorus* Williston, 1910 (Temnospondyli: Dissorophidae), from the Lower Permian of Texas. *Journal of Vertebrate Paleontology* 40:e1776720. DOI: 10.1080/02724634.2020.1776720

Anderson JS. 2005. On the skull of *Cacops aspidephorus* Williston (Tetrapoda; Temnospondyli; Dissorophidae) from the Lower Permian of Texas. Part 1: lower jaw anatomy. *New Mexico Museum of Natural History & Science Bulletin* 30:15.

Berman DS, Henrici AC, Brezinski DK, Kollar AD. 2010. A new trematopid amphibian (Temnospondyli: Dissorophoidea) from the Upper Pennsylvanian of western Pennsylvania: earliest record of terrestrial vertebrates responding to a warmer, drier climate. *Annals of Carnegie Museum* 78:289–319. DOI: 10.2992/007.078.0401

Berman DS, Henrici AC, Martens T, Sumida SS, Anderson JS. 2011. *Rotaryus gothae*, a new trematopid (Temnospondyli: Dissorophoidea) from the Lower Permian of Central Germany. *Annals of Carnegie Museum* 80:49–66. DOI: 10.2992/007.080.0106

Berman DS, Lucas SG. 2003. *Aspidosaurus binasser* (Amphibia, Temnospondyli), a new species of Dissorophidae from the Lower Permian of Texas. *Annals of Carnegie Museum* 72:241–262.

Berman DS, Reisz R, Eberth DA. 1985. *Ecolsonia cutlerensis*, an Early Permian dissorophid amphibian from the Cutler Formation of north-central New Mexico. *New Mexico Bureau of Mines and Minerals Research Circular* 191:1–31.

Berman DS, Reisz RR, Eberth DA. 1987. A new genus and species of trematopid amphibian from the Late Pennsylvanian of north-central New Mexico. *Journal of Vertebrate Paleontology* 7:252–269. DOI: 10.1080/02724634.1987.10011659

Berman DS, Reisz RR, Fracasso, MA. 1981. Skull of the Lower Permian dissorophid amphibian *Platyhystrix rugosus*. *Annals of Carnegie Museum* 50:391–416.

Bolt JR. 1969. Lissamphibian origins: possible protolissamphibian from the Lower Permian of Oklahoma. *Science* 166:888–891. DOI: 10.1126/science.166.3907.888

Bolt JR. 1974a. A trematopsid skull from the Lower Permian, and analysis of some characters of the dissorophoids (Amphibia, Labyrinthodontia) otic notch. *Fieldiana: Geology* 30:67–79.

Bolt JR. 1974b. Evolution and functional interpretation of some suture patterns in Paleozoic labyrinthodont amphibians and other lower tetrapods. *Journal of Paleontology* 48:434–458.

Boy JA. 1995. Über die Micromelerpetontidae (Amphibia: Temnospondyli). 1. Morphologie und Paläoökologie des *Micromelerpeton credneri* (Unter-Perm; SW-Deutschland). *Paläontologische Zeitschrift* 69:429–457. DOI: 10.1007/BF02987805

Brazeau MD, Guillerme T, Smith MR. 2019. An algorithm for morphological phylogenetic analysis with inapplicable data. *Systematic Biology* 68:619–631. DOI: 10.1093/sysbio/syy083

Carroll RL. 1964a. Early evolution of the dissorophid amphibians. *Bulletin of the Museum of Comparative Zoology* 131:161–250.

Carroll RL. 1964b. The relationships of the rhachitomous amphibian Parioxys. American Museum Novitates 2167:1–11.

Clack JA, Milner AR. 2009. Morphology and systematics of the Pennsylvanian amphibian *Platyrhinops lyelli* (Amphibia: Temnospondyli). *Earth and Environmental Science Transactions of the Royal Society of Edinburgh* 100:275–295. DOI: 10.1017/S1755691010009023

Case EC, Williston SW. 1913. A description of *Aspidosaurus novomexicanus*. Carnegie Institute Washington 181: 7–9.

Daly E. 1994. The Amphibamidae (Amphibia: Temnospondyli), with a description of a new genus from the Upper Pennsylvanian of Kansas. *Miscellaneous Publication of the University of Kansas Museum of Natural History* 85: 1–59.

DeMar RE. 1967. Two new species of Broiliellus (Amphibians) from the Permian of Texas. *Fieldiana: Geology* 16:117–129.

Dilkes D, Brown LE. 2007. Biomechanics of the vertebrae and associated osteoderms of the Early Permian amphibian *Cacops aspidephorus*. Journal of Zoology 271:396–407. DOI: 10.1111/j.1469-7998.2006.00221.x

Dilkes DW. 1990. A new trematopsid amphibian (Temnospondyli: Dissorophoidea) from the Lower Permian of Texas. *Journal of Vertebrate Paleontology* 10:222–243. DOI: 10.1080/02724634.1990.10011809

Dilkes DW. 1993. Biology and evolution of the nasal region in trematopid amphibians. *Palaeontology* 36:839–853.

Dilkes DW. 2009. Comparison and biomechanical interpretations of the vertebrae and osteoderms of *Cacops aspidephorus* and *Dissorophus multicinctus* (Temnospondyli, Dissorophidae). *Journal of Vertebrate Paleontology* 29:1013–1021. DOI: 10.1671/039.029.0410

Dilkes DW. 2020. Revision of the Early Permian dissorophid '*Dissorophus' angustus* (Temnospondyli: Dissorophoidea). *Journal of Vertebrate Paleontology* 40:e1801704. DOI: 10.1080/02724634.2020.1801704

Efremov IA. 1937. On the Permo-Triassic labyrinthodonts from USSR. IV. A note on the lost forms *Zygosaurus* Eichwald and *Chalcosaurus* von Meyer. *Trudy Paleontologichesko Instituta Akademiyi Nauk SSSR* 8:17–27. [in Russian]

Eichwald ED. 1848. Über die Saurier des kupferführenden Zechsteins Russlands. *Bulletin de la Société Impériale des naturalistes de Moscou* 21:136–204. [in German]

Fröbisch NB, Brar A, Reisz RR. 2015. New specimen of *Cacops woehri* indicates differences in the ontogenetic trajectories among cacopine dissorophids. *Fossil Record* 18:73–80. DOI: 10.5194/fr-18-73-2015

Fröbisch NB, Reisz RR. 2008. A new Lower Permian amphibamid (Dissorophoidea, Temnospondyli) from the fissure fill deposits near Richards Spur, Oklahoma. Journal of Vertebrate Paleontology 28:1015–1030. DOI: 10.1671/0272-4634-28.4.1015

Fröbisch NB, Reisz RR. 2012. A new species of dissorophid (*Cacops woehri*) from the Lower Permian Dolese quarry, near Richards Spur, Oklahoma. *Journal of Vertebrate Paleontology* 32:35–44. DOI: 10.1080/02724634.2012.633586

Gee BM, Bevitt JJ, Reisz RR. 2019. Dissorophid diversity at the early Permian cave system near Richards Spur, Oklahoma. *Palaeontologica Electronica* 22.2.46A:1–32. DOI: 10.26879/976

Gee BM, Reisz RR. 2018a. Cranial and postcranial anatomy of *Cacops morrisi*, a eucacopine dissorophid from the early Permian of Oklahoma. *Journal of Vertebrate Paleontology* 38:e1433186. DOI: 10.1080/02724634.2018.1433186

Gee BM, Reisz RR. 2018b. Postcrania of large dissorophid temnospondyls from Richards Spur, Oklahoma. *Fossil Record* 21:79–91. DOI: 10.5194/fr-21-79-2018

Gee BM. 2020. Size matters: the effects of ontogenetic disparity on the phylogeny of Trematopidae (Amphibia: Temnospondyli). *Zoological Journal of the Linnean Society*, 190:79-113. DOI: 10.1093/zoolinnean/zlz170

Gee BM, Berman DS, Henrici AC, Pardo JD, Huttenlocker AK. 2021. New information on the dissorophid *Conjunctio* (Temnospondyli) based on a specimen from the Cutler Formation of Colorado, U.S.A. *Journal of Vertebrate Paleontology* 40:e1877152. DOI: 10.1080/02724634.2020.1877152

Gubin YM. 1980. [New Permian dissorophids of the Ural forelands]. *Paleontologicheskii Zhurnal* 1980:82–90. [in Russian]

Gubin YM. 1987. On the systematic position and age of some labyrinthodonts from the Upper Permian deposits of the region west of the Urals. *Paleontologicheskii Zhurnal* 1987:94–99. [in Russian]

Holmes R, Berman DS, Anderson JS, 2013. A new dissorophid (Temnospondyli, Dissorophoidea) from the Early Permian of New Mexico (United States). *Comptes Rendus Palevol* 12:419–435. DOI: 10.1016/j.crpv.2013.07.002

Langston W. 1953. Permian amphibians from New Mexico. *University of California Publications in Geological Sciences* 29:349–416.

Li J-L, Cheng Z-W. 1999. New anthracosaur and temnospondyl amphibians from Gansu, China – the fifth report on Late Permian Dashankou lower tetrapod fauna. *Vertebrata PalAsiatica* 37:234–247.

Liu J. 2018. Osteology of the large dissorophid temnospondyl *Anakamacops petrolicus* from the Guadalupian Dashankou Fauna of China. *Journal of Vertebrate Paleontology*: 38:e1513407. DOI: 10.1080/02724634.2018.1513407

Maddin HC, Fröbisch NB, Evans DC, Milner AR. 2013. Reappraisal of the Early Permian amphibamid *Tersomius texensis* and some referred material. *Comptes Rendus Palevol* 12:447–461. DOI: 10.1016/j.crpv.2013.06.007

Milner AR. 1985. On the identity of *Trematopsis seltini* (Amphibia: Temnospondyli) from the Lower Permian of Texas. *Neues Jahrbuch für Geologie und Paläontologie-Monatshefte* 1985:357–367. DOI: 10.1127/njgpm/1985/1985/357

Milner AR. 2018 (for 2019). Two primitive trematopid amphibians (Temnospondyli, Dissorophoidea) from the Upper Carboniferous of the Czech Republic. *Earth and Environmental Science Transactions of the Royal Society of Edinburgh* 109:201–223. DOI: 10.1017/S1755691018000725

Olson EC. 1941. The family Trematopsidae. *The Journal of Geology* 49:149–176. DOI: 10.1086/624952

Pawley K, Warren A. 2006. The appendicular skeleton of *Eryops megacephalus* (Temnospondyli: Eryopoidea) from the lower Permian of North America. Journal of Paleontology 80:561–580. DOI: 10.1666/0022-3360(2006)80[561:TASOEM]2.0.CO;2

Polley BP, Reisz RR. 2011. A new Lower Permian trematopid (Temnospondyli: Dissorophoidea) from Richards Spur, Oklahoma. *Zoological Journal of the Linnean Society* 16:789–815. DOI: 10.1111/j.1096-3642.2010.00668.x

Reisz RR, Schoch RR, Anderson JS. 2009. The armoured dissorophid *Cacops* from the Early Permian of Oklahoma and the exploitation of the terrestrial realm by amphibians. *Naturwissenschaften* 96:789. DOI: 10.1007/s00114-009-0533-x

Schoch RR, Milner AR. 2014. Handbook of Paleoherpetology Part 3A2. Temnospondyli I. München: Verlag Dr. Friedrich Pfeil.

Schoch RR, Rubidge BS. 2005. The amphibamid *Micropholis* from the Lystrosaurus assemblage zone of South Africa. *Journal of Vertebrate Paleontology* 25:502–522. DOI: 10.1671/0272-4634(2005)025[0502:TAMFTL]2.0.CO;2

Schoch RR, Sues H-D. 2013. A new dissorophid temnospondyl from the Lower Permian of north-central Texas. *Comptes Rendus Palevol* 12:437–445. DOI: 10.1016/j.crpv.2013.04.002

Schoch RR. 1999. Studies on braincases of early tetrapods: structure, morphological diversity, and phylogeny-2. Dissorophoids, eryopids, and stereospondyls. *Neues Jahrbuch für Geologie und Paläontologie-Abhandlungen* 213:289–312. DOI: 10.1127/njgpa/213/1999/289

Schoch RR. 2012. Character distribution and phylogeny of the dissorophid temnospondyls. *Fossil Record* 15:121–137. DOI: 10.1002/mmng.201200010

Schoch RR. 2013. The evolution of major temnospondyl clades: an inclusive phylogenetic analysis. *Journal of Systematic Palaeontology* 11:73–705. DOI: 10.1080/14772019.2012.699006

Schoch RR. 2018 (for 2019). The putative lissamphibian stem-group: phylogeny and evolution of the dissorophoid temnospondyls. *Journal of Paleontology* 93:137–156. DOI: 10.1017/jpa.2018.67

Sigurdsen T, Bolt JR. 2010. The Lower Permian amphibamid *Doleserpeton* (Temnospondyli: Dissorophoidea), the interrelationships of amphibamids, and the origin of modern amphibians. *Journal of Vertebrate Paleontology* 30:1360–1377. DOI: 10.1080/02724634.2010.501445

Sullivan C, Reisz RR, May WJ. 2000. Large dissorophoid skeletal elements from the Lower Permian Richards Spur fissures, Oklahoma, and their paleoecological implications. *Journal of Vertebrate Paleontology* 20:456–461. DOI: 10.1671/0272-4634(2000)020[0456:LDSEFT]2.0.CO;2

Sumida SS, Berman DS, Martens T. 1998. A new trematopid amphibian from the Lower Permian of central Germany. *Palaeontology* 41:605–630.

Williston SW. 1909. New or little-known Permian vertebrates: *Trematops*, new genus. The Journal of Geology 17:636–658. DOI: 10.1086/621665

Williston SW. 1910. *Cacops, Desmospondylus*; new genera of Permian vertebrates. *Bulletin of the Geological Society of America* 21:249–284. DOI: 10.1130/GSAB-21-249

Williston SW. 1914. *Broiliellus*, a new genus of amphibians from the Permian of Texas. *The Journal of Geology* 22:49–56.
